# Interaction analysis of non‐bacterial respiratory pathogens during and after the coronavirus disease 2019 pandemic in two cities along the eastern coast of China

**DOI:** 10.1002/ped4.70034

**Published:** 2026-01-19

**Authors:** Wanxian Ye, Jishan Zheng, Yungang Yang, Xinyue Song, Xiang Yuan, Lan Yang, Jian Yu, Hailin Zhang, Shunhang Wen

**Affiliations:** ^1^ Department of Pediatric Respiratory Medicine National Key Clinical Specialty of Pediatric Respiratory Medicine Institute of Pediatrics The Second Affiliated Hospital and Yuying Children's Hospital of Wenzhou Medical University Wenzhou Zhejiang China; ^2^ Pediatric Respiratory Department Ningbo Women and Children's Hospital of Ningbo University Ningbo Zhejiang China; ^3^ Department of Pediatrics the First Affiliated Hospital of Xiamen University Xiamen Fujian China; ^4^ Department of Clinical Laboratory The Second Affiliated Hospital and Yuying Children's Hospital of Wenzhou Medical University Wenzhou Zhejiang China

**Keywords:** Children, COVID‐19 pandemic, Non‐bacterial respiratory pathogens, Pathogen, Vector autoregressive model

## Abstract

**Importance:**

The coronavirus disease 2019 (COVID‐19) pandemic and associated non‐pharmaceutical interventions (NPIs) have significantly altered the epidemiology of respiratory pathogens. Understanding the interactions between non‐bacterial respiratory pathogens is crucial for clinical management and surveillance.

**Objective:**

To investigate how interactions among non‐bacterial respiratory pathogens in children changed during and after the COVID‐19 pandemic in two cities in eastern China.

**Methods:**

This retrospective study reviewed the data of children hospitalized with acute respiratory tract infections in Wenzhou and Ningbo between March 1, 2021, and February 28, 2024. The SureX 13 respiratory pathogen multiplex kit was used to detect 13 pathogen types/subtypes in the respiratory tract secretion specimens. The chi‐square test or Fisher's exact test, virus correlation, and vector autoregressive modelling were employed to evaluate correlations and dynamic changes in the weekly positive detection rates before and after the NPIs.

**Results:**

Among the 73 096 children tested, 65.18% had at least one non‐bacterial respiratory pathogen, and 11.97% had multiple pathogens. Detection rates declined significantly by 56.65% during NPIs but rebounded by 75.46% afterward, particularly for *Mycoplasma pneumoniae*, which increased from 5.29% to 34.78%. Post‐pandemic, the co‐detection of non‐bacterial respiratory pathogens increased, with interaction patterns varying by phase. Notably, after the pandemic, the positive and negative correlations among pathogens intensified, with a significant increase in negative associations. Furthermore, a persistent negative correlation existed between the influenza B virus and *Mycoplasma pneumoniae* (−0.36 to −0.25), suggesting the potential presence of pathogen interference.

**Interpretation:**

The interactions between non‐bacterial respiratory pathogens markedly changed after COVID‐19, showing strengthened correlations, which were primarily negative in nature. These observations underscore the importance of the ongoing surveillance of respiratory pathogens in evolving NPIs and epidemiological patterns.

## Graphical abstract

What is Known

• Co‐infections can influence pathogen replication, disease severity, and the duration of illness.

• Co‐infection patterns and temporal correlations have been characterized among various respiratory viruses and atypical pathogens.

What is New

• A consistent negative correlation between influenza B and Mycoplasma pneumoniae suggests potential pathogen interference.

• The coronavirus disease 2019 pandemic and related non‐pharmaceutical interventions significantly altered the epidemiological trends of respiratory pathogens.

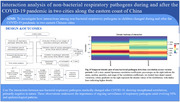

## INTRODUCTION

Acute respiratory infections are the leading cause of hospital admissions in children.^1^ Multiplex reverse transcription polymerase chain reaction (PCR) has improved the detection rates of respiratory tract pathogens, highlighting the role of multiple non‐bacterial co‐infections in the pathogenesis of respiratory diseases.^2^, ^3^, ^4^ Co‐infections may alter pathogen replication dynamics, thereby influencing symptom severity and illness duration.^5^, ^6^, ^7^ The epidemiological patterns of non‐bacterial respiratory pathogens suggest potential interrelationships in pediatric populations. Numerous non‐bacterial respiratory pathogens do not transmit in isolation; instead, they interact with one another during their transmission processes. The activity level of a particular virus can affect the emergence of another. Furthermore, the severe acute respiratory syndrome coronavirus 2 (SARS‐CoV‐2) pandemic has significantly modified the transmission dynamics of various viruses. However, the extent of this interaction remains unclear. During the coronavirus disease 2019 (COVID‐19) pandemic, various respiratory viruses, including *Mycoplasma pneumoniae*, exhibited distinct epidemiological trends from those of previous years.^8^ In the aftermath of the COVID‐19 pandemic, there has been a resurgence of *M. pneumoniae*, leading to outbreaks in various regions worldwide. These changes are attributed to non‐pharmaceutical interventions (NPIs) implemented to mitigate the spread of COVID‐19 and to the intricate interactions among different pathogens.^9^, ^10^, ^11^, ^12^

Owing to their relatively immature immune systems, children are more susceptible to non‐bacterial respiratory infections.^1^ Research on the interactions between these pathogens and their effects during the COVID‐19 pandemic is essential for improving diagnosis, treatment, and prevention strategies in pediatric populations. The post‐pandemic re‐emergence of pathogens, such as respiratory syncytial virus (RSV) and influenza, in conjunction with the ongoing circulation of SARS‐CoV‐2, highlights the intricate nature of their interactions. Investigating the interplay among these pathogens and their impact during the COVID‐19 pandemic is crucial for advancing diagnostic, therapeutic, and preventive measures in pediatric populations. Gaining a more profound understanding of how one infection modulates susceptibility to another will directly inform public health strategies regarding outbreak preparedness and allocation of resources for pediatric care.

In this study, we aimed to examine the interactions among non‐bacterial respiratory pathogens during and after the COVID‐19 pandemic in two cities in eastern China. Time‐series data were assessed after controlling for confounding variables to evaluate the correlations between these pathogens and their interaction characteristics.

## METHODS

### Ethics approval

This study was approved by the Ethics Committees of the Second Affiliated Hospital and Yuying Children's Hospital, Wenzhou Medical University (2024K24401). Informed consent was waived by our ethics committee because of the retrospective nature of the study.

### Patient and sample information

This retrospective study was conducted from March 1, 2021, to February 28, 2024, and included hospitalized children with acute respiratory tract infections in the Second Affiliated Hospital and Yuying Children's Hospital, Wenzhou Medical University, and Ningbo Women and Children's Hospital of Ningbo University. All pediatric patients underwent routine testing for non‐bacterial respiratory pathogens. Sputum or nasopharyngeal samples were collected according to clinically standardized protocols and sent to the laboratory for PCR‐capillary electrophoresis fragment analysis using the SureX 13 Respiratory Pathogen Multiplex Detection Kit (Health Gene Technologies, Inc., Ningbo, China). The pathogens tested included influenza A virus (Inf A), H1N1 (2009), seasonal H3N2, influenza B virus (Inf B), human parainfluenza virus (HPIV), human RSV (HRSV), human adenovirus (HAdV), human rhinovirus (HRV), human metapneumovirus (HMPV), bocavirus, coronavirus (including OC43, 229E, NL63, and HKU1), *Chlamydia*, and *M. pneumoniae*. The detection rate of *Chlamydia* pneumonia was substantially low, with no statistical significance. Therefore, it was not shown in the figures and tables. Patient age and specimen collection dates were retrieved from the hospital database for statistical analysis.

### Statistical analysis

This study divided the years into 53 weeks, and the detection rate of non‐bacterial pathogens was determined by dividing the number of positive cases per week by the total number of tests conducted. The weekly distribution of each non‐bacterial pathogen was organized according to percentiles ranging from 0 to 1. The Chi‐square or Fisher's exact test was employed to compare the positive detection rates of pathogens, with a bilateral *P*‐value of 0.05 as the statistical significance threshold. The four seasons were defined based on Chinese solar terms: spring (weeks 13–25), summer (weeks 26–38), autumn (weeks 39–51), and winter (weeks 52–12 of the subsequent year).

In accordance with the modifications to China’s epidemic prevention and control policies, as well as the directives outlined in the ‘Guidelines for the Public to Wear Masks to Prevent Novel Coronavirus Infection (April 2023 Edition)’ (https://www.gov.cn/xinwen/gwylflkjz217/wzsl.htm; https://www.gov.cn/lianbo/2023‐04/12/content_5751073.htm), two critical time points were established: the cessation of epidemic control measures (week 50 of 2022) and the discontinuation of stringent mask‐wearing by the public (week 15 of 2023). The study was categorized into three phases: Phase 1 (9th week of 2021 to the 50th week of 2022) preceded lifting epidemic restrictions, with the national action plan in effect; Phase 2 (51st week of 2022 to the 14th week of 2023) encompassed the acute COVID‐19 pandemic period, characterized by the gradual relaxation of the national action plan and cessation of stringent mask‐wearing mandates; and Phase 3 (from the 15th week of 2023) began post‐pandemic, marked by school reopening and resumption of social activity.

All statistical analyses were conducted using R version 4.3.1 (R Foundation for Statistical Computing, Vienna, Austria).

#### Correlation analysis

A simple bivariate cross‐correlation analysis based on weekly pathogen detection rates was performed to quantitatively assess the relationships between respiratory pathogens. Spearman's rank correlation coefficient was calculated using the cor() function in R version 4.3.1 (with statistical significance defined as *P* < 0.05). The Benjamini‐Hochberg method was used to control the false discovery rate. The probability of the co‐detection of each pathogen with other pathogens was calculated at the individual level.

#### Vector autoregressive model

To distinguish genuine interactions among pathogens from correlations, we employed a vector autoregressive (VAR) model using the Vars package (v.1.6.1) and time‐series data from multiple contemporaneous pathogens, as represented by Eq. (1):

(1) $$\mathrm{yt}=A1\;y\left\{t-1\right\}+\text{…}+\mathrm{Ap}\;y\left\{t-p\right\}+C\;\mathrm{Dt}+\mathrm{ut}$$

where yt is a k‐dimensional endogenous variable representing the probability of weekly detection rates of the 11 pathogens (k = 11). The yt value was calculated as the first‐order difference. Dt denotes an exogenous variable, including age, sex, and season. The *p* represents the lag phase, which is determined as lag = 1 based on the Akaike Information Criterion (AIC) value. C represents estimation through a matrix. ut is a white noise process with a mean of zero. Matrices A and B are the parameter matrices to be estimated, whereas *t* signifies a random disturbance assumed to follow a multivariate normal distribution with a mean of zero and a covariance matrix R of 11 × 11. The Augmented Dickey–Fuller test indicates that the series is stationary. Impulse response and variance decomposition analyses were conducted, and the residual correlation matrix inferred the interactions among pathogens.

## RESULTS

### Detection of respiratory non‐bacterial pathogens

Among 73 096 included pediatric patients, 47 641 (65.18%) had at least one pathogen detected, and 8746 (11.97%) harbored multiple pathogens. *M. pneumoniae* was the most frequently detected single pathogen, identified in 11 676 cases (15.97%). HRV was the most prevalent virus, found in 8242 patients (11.28%), followed by HRSV in 5450 (7.46%), HMPV in 2717 (3.72%), HAdV in 2600 (3.56%), and HPIV in 2413 (3.30%). Detection rates declined significantly by 56.65% during NPIs but rebounded by 75.46% afterward, particularly for *M. pneumoniae*, which increased from 5.29% to 34.78%.

**FIGURE 1:**
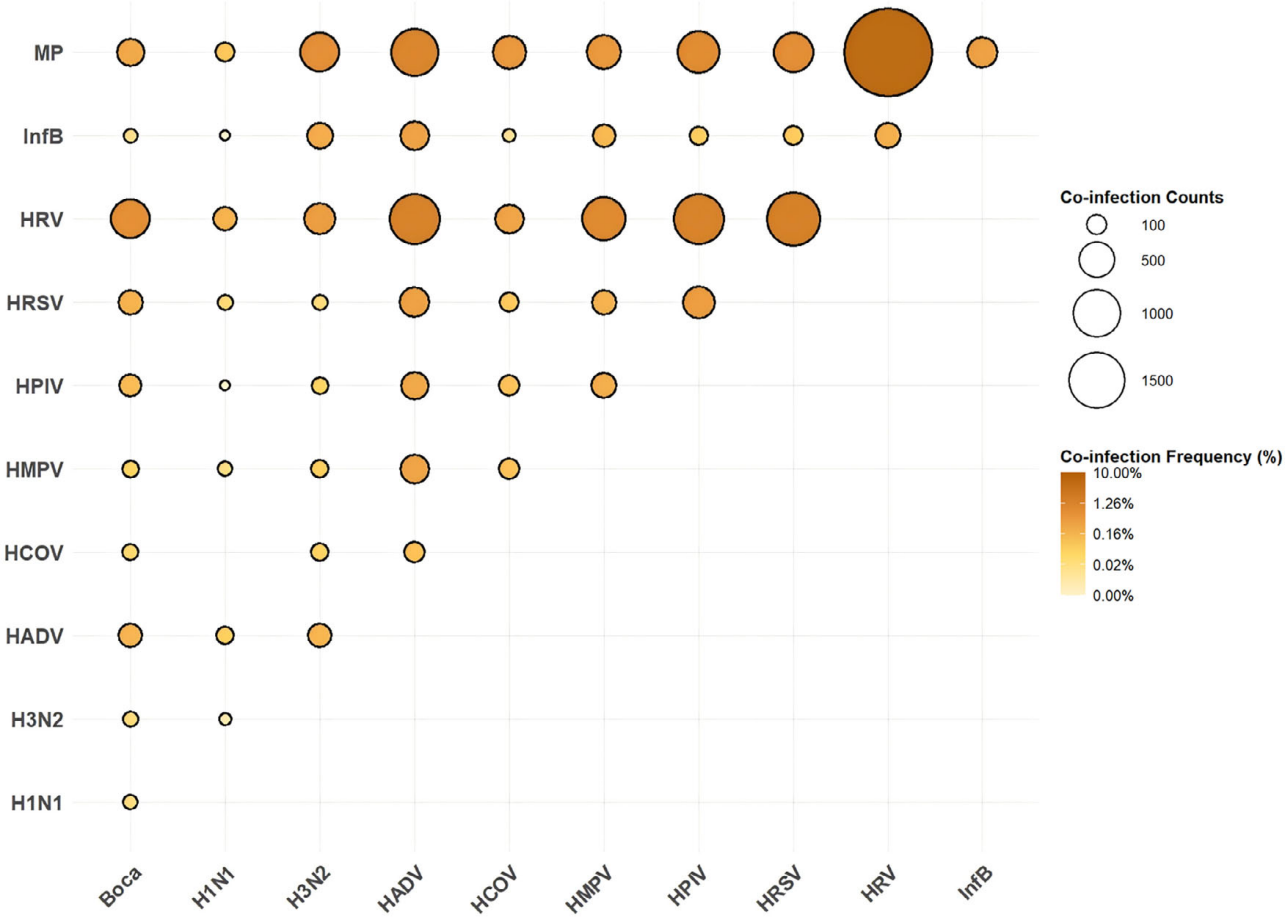
Frequency of confirmed co‐infection for each pair of respiratory pathogens across two cities from the 9th week of 2021 to the 9th week of 2024. Frequency is represented by dot size; colour indicates the proportion of each co‐infection pair among all tested samples. Boca, bocavirus; InfB, influenza B virus; HPIV, human parainfluenza virus; HRSV, human respiratory syncytial virus; HAdV, human adenovirus; HRV, human rhinovirus; HMPV, human metapneumovirus; HCOV, human coronavirus; MP, *Mycoplasma pneumoniae*.

Concurrent examinations of multiple pathogens revealed a higher prevalence of viruses, including HRV (*n* = 5329, 7.29%) and *M. pneumoniae* (*n* = 4382, 5.99%). The combination of HRV and *M. pneumoniae* (*n* = 1897, 2.60%) was the most frequently detected coinfection, whereas HRV and HRSV (*n* = 636, 0.87%) were the most common viral coinfections (Figure 1).

Given that Wenzhou and Ningbo share a subtropical monsoon climate and exhibit highly similar geographical and climatic characteristics (with only slight differences in mean annual temperature and relative humidity), and that a unified research protocol was used across cooperative centres in both cities, we primarily employed pooled data in our analysis to enhance statistical power. The analysis of cross‐city heterogeneity is presented in Tables S1 and S2.

**FIGURE 2:**
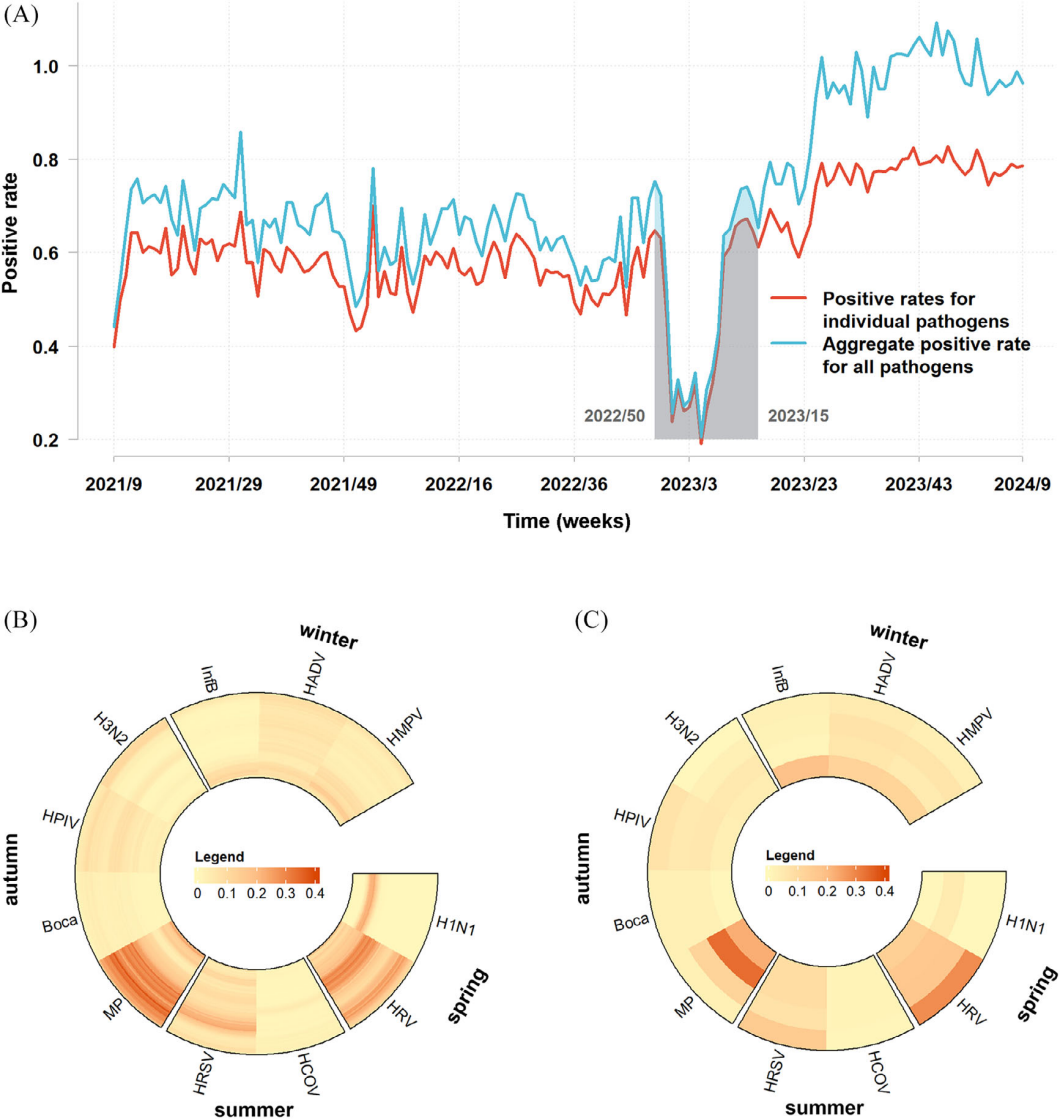
Temporal distribution of viral pathogens associated with acute respiratory infections in Wenzhou and Ningbo from the 9th week of 2021 to the 9th week of 2024. (A) Weekly positive rates for individual respiratory pathogens and the aggregate positive rate for all pathogens; shaded period: week 50 of 2022 to week 15 of 2023. The increasing area between the two lines corresponds to a gradual increase in co‐detections. (B) Weekly average virus detection rates, categorised by percentiles from 0 to 0.4. The legend that values above 0.4 are not represented. (C) Thermograms displaying annual virus detection rates, sorted by percentiles from 0 to 0.4. The legend that values above 0.4 are not represented. Boca, bocavirus; Inf B, influenza B virus; HPIV, human parainfluenza virus; HRSV, human respiratory syncytial virus; HAdV, human adenovirus; HRV, human rhinovirus; HMPV, human metapneumovirus; HCOV, human coronavirus; MP, *Mycoplasma pneumoniae*.

The overall detection rate of respiratory pathogens fluctuated weekly from 19.21% to 82.83% (Figure 2A). Respiratory pathogens were subjectively categorised into four groups based on their peak season (Figure 2B, C). The first group, comprising H1N1 and HRV, peaked primarily in the spring. The second group, HRSV and coronavirus, was predominant in the summer. The third group, comprising *M. pneumoniae*, bocavirus, HPIV, and H3N2, exhibited marginally higher detection rates in autumn. The fourth group, Inf B, HMPV, and HAdV, was predominant in winter.

### Differences in pathogen prevalence during and after the pandemic

**FIGURE 3:**
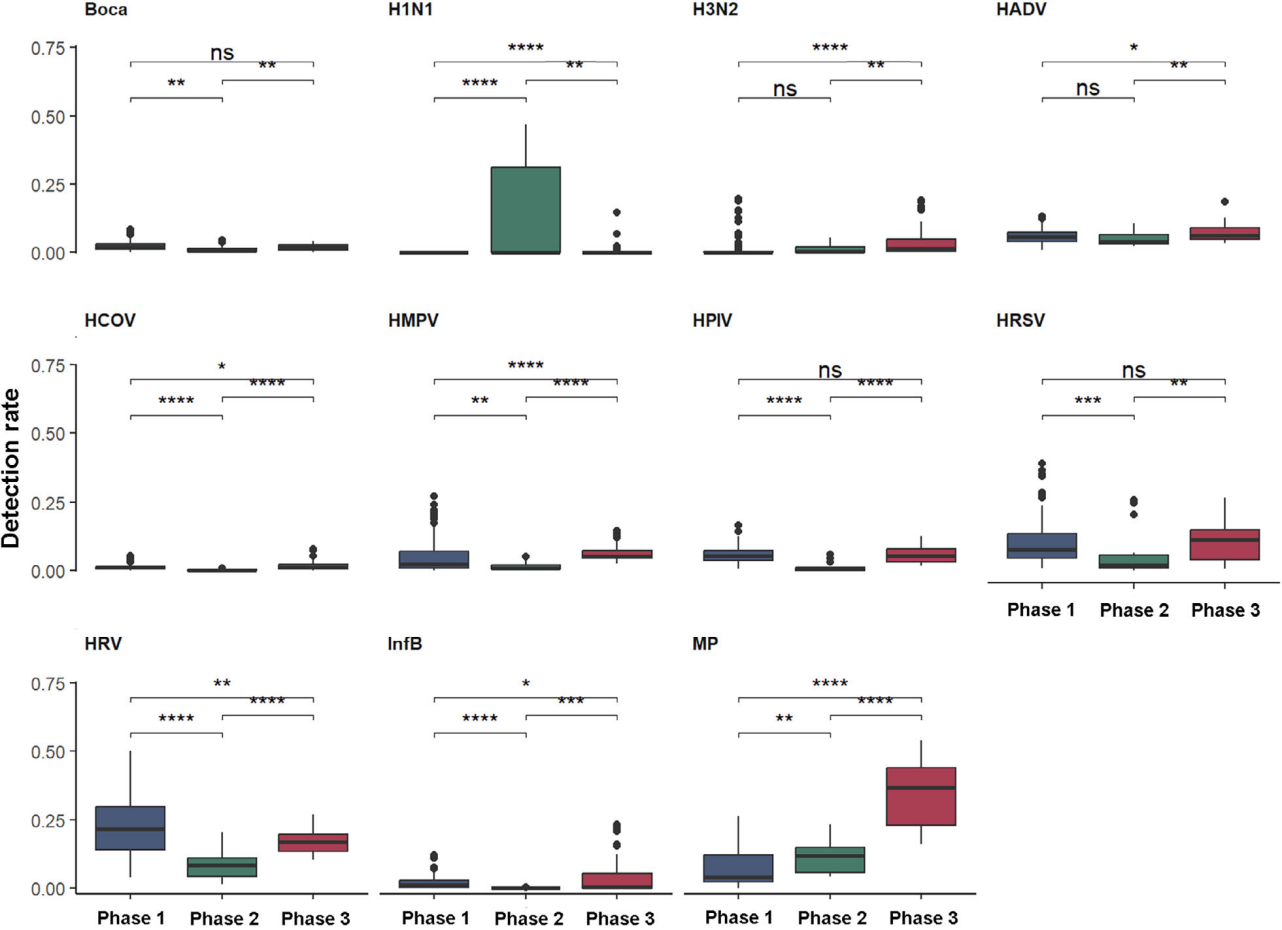
Infection rates of various pathogens across three distinct phases. Phase 1: 9th week of 2021 to 50th week of 2022; Phase 2: 51st week of 2022 to 14^th^ week of 2023; Phase 3: from the 15^th^ week of 2023 onward. ^*^ *P* < 0.05; ^**^ *P* < 0.01; ^***^ *P* < 0.001; ^****^ *P* < 0.0001. Boca, bocavirus; InfB, influenza B virus; HPIV, human parainfluenza virus; HRSV, human respiratory syncytial virus; HAdV, human adenovirus; HRV, human rhinovirus; HMPV, human metapneumovirus; HCOV, human coronavirus; MP, *Mycoplasma pneumoniae*; ns, not significant.

The detection rates of pathogens across the three phases revealed that during Phase 2, when large‐scale NPIs were partially relaxed, H1N1 influenza infections exhibited a significant upward trend (*P* < 0.001), whereas the detection rates of H3N2 influenza (*P* = 0.935) and HAdV (*P* = 0.125) remained comparable to those of Phase 1. Concurrently, the detection rates of other viruses declined significantly. In Phase 3, characterized by the lifting of NPIs, the detection rates of most non‐bacterial respiratory pathogens rebounded, surpassing those in Phase 1. Notably, the detection rate of *M. pneumoniae* in Phase 3 significantly exceeded that in Phases 1 and 2. Conversely, the detection rates of bocavirus, HPIV, and HRSV in Phase 3 did not differ significantly from the pandemic levels in Phases 1 (Figure 3).

**FIGURE 4:**
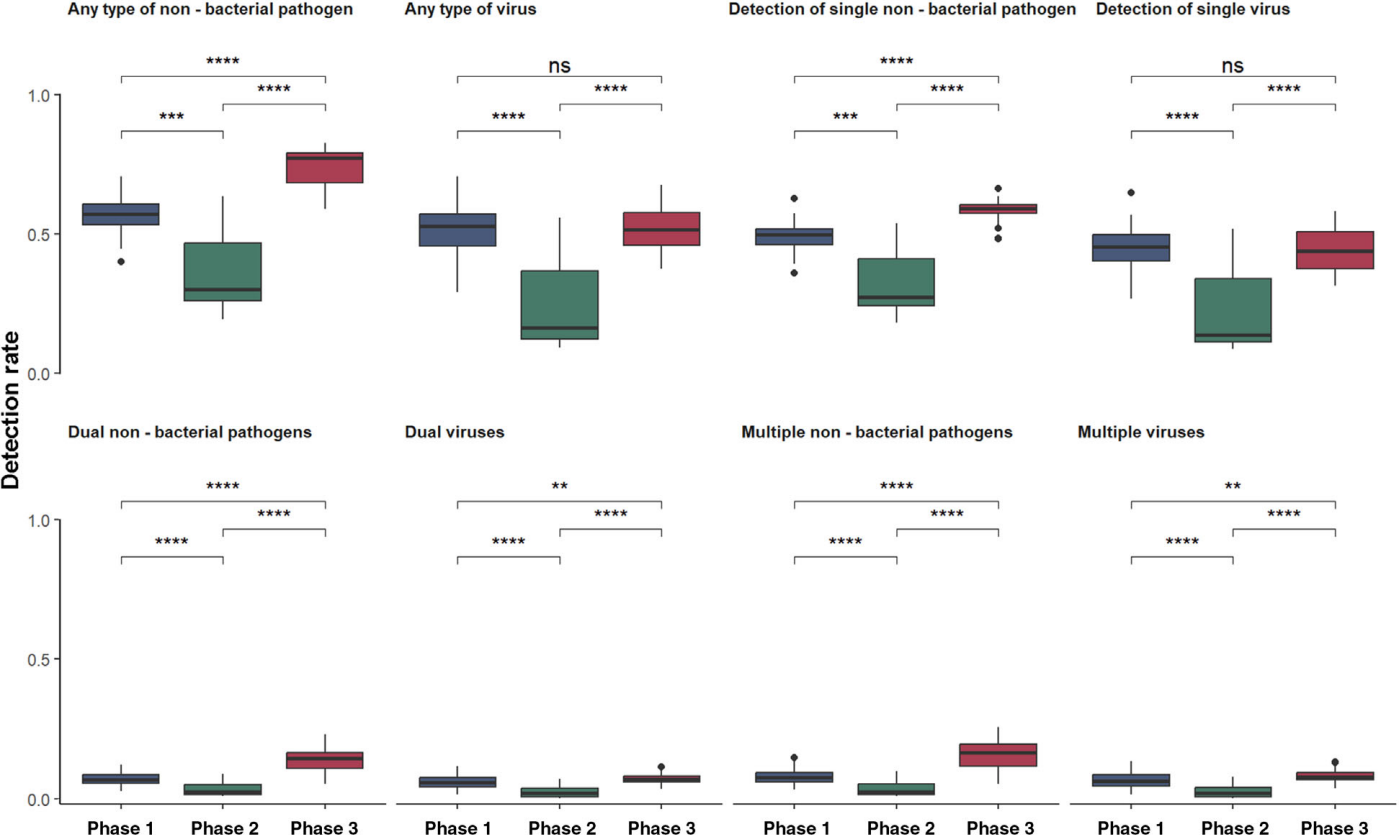
Variation in virus and non‐bacterial pathogen detection rates across three distinct phases. ^**^ *P* < 0.01; ^***^ *P* < 0.001; ^****^ *P* < 0.0001. ns, not significant.

Overall, the co‐detection rates differed significantly across the phases (Figure 4). Specifically, the mixed detection rate in Phase 2 was significantly lower than that in the other phases and the detection rate in Phase 3 was significantly higher than that in Phase 1. Given that the *M. pneumoniae* outbreak was lifted after restrictions were lifted, its detection was excluded to focus on changes in viral detection. The detection rate of two or more viruses was lowest in Phase 2 (*P* < 0.0001) and significantly higher in Phase 3 than in Phase 1 (*P* < 0.01). No significant differences were observed in any individual virus between Phases 1 and 3 (*P* = 0.796).

### Correlations between non‐bacterial respiratory pathogens

**FIGURE 5:**
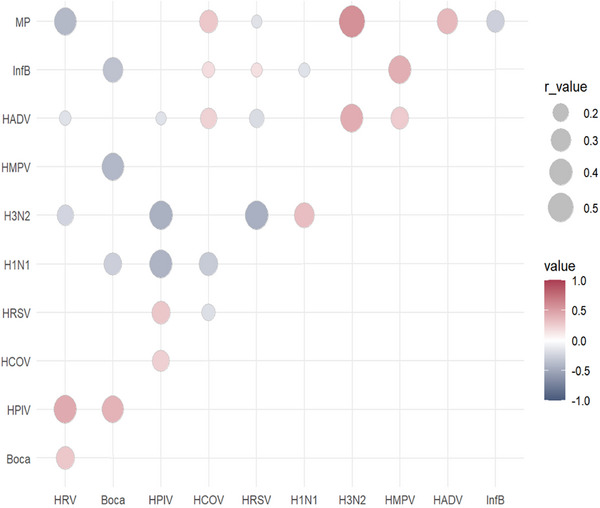
Correlations of respiratory pathogens at the population level in Ningbo and Wenzhou from Week 9 of 2021 to Week 9 of 2024. The interrelationships among common respiratory pathogens are presented; blue–gray circles indicate negative correlations, red circles indicate positive correlations, and larger/darker circles represent stronger correlations. All correlations depicted are statistically significant, as determined by Spearman's rank correlation (*P* < 0.05). Boca, bocavirus; Inf B, influenza B virus; HPIV, human parainfluenza virus; HRSV, human respiratory syncytial virus; HAdV, human adenovirus; HRV, human rhinovirus; HMPV, human metapneumovirus; HCOV, human coronavirus; MP, *Mycoplasma pneumoniae*.

Spearman's rank correlation analysis revealed 16 negative and 15 positive correlations among respiratory pathogens (Figure 5). The HPIV, *M. pneumoniae*, HAdV, H3N2, and Inf B demonstrated the strongest correlation with other pathogens. In particular, HPIV were significantly positively correlated with HRV (*r* = 0.43, *P* < 0.001), HRSV (*r* = 0.29, *P* < 0.001), and bocavirus (*r* = 0.39, *P* < 0.001), and negatively correlated with H3N2 (*r* = −0.45, *P* < 0.001), H1N1 (*r* = −0.43, *P* < 0.001), and HAdV (*r* = −0.16, *P* = 0.037). *M. pneumoniae* exhibited notable correlations with H3N2 (*r* = 0.57, *P* < 0.001), HAdV (*r* = 0.39, *P* < 0.001), and HCOV (*r* = 0.27, *P* < 0.001), and was negatively correlated with HRV (*r* = −0.40, *P* < 0.001) and Inf B (*r* = −0.26, *P* < 0.001). Additionally, H3N2 was negatively correlated with HRV (*r* = −0.23, *P* = 0.002) and HRSV (*r* = −0.45, *P* < 0.001), whereas H1N1 was negatively correlated with bocavirus (*r* = −0.27, *P* < 0.001) and HCOV (*r* = −0.29, *P* < 0.001). To facilitate a more accurate assessment, we supplemented the analysis with individual correlation maps for 16 sets of negative and 15 sets of positive pathogen pairs (Figures S1–S31).

VAR modelling distinguishes genuine pathogen interactions from correlations by examining the lagged effects of pathogens on each other using impulse response functions. The unit root test confirmed that all 11 pathogens were stationary. When the lag period was set to 1, the AIC reached its minimum value (lag1: −86.606; lag2: −86.087; lag3: −85.503; lag4: −85.404), indicating the optimal model fit. The correlation matrix of residuals indicated a negative interaction between the Inf B and *M. pneumoniae* (correlation coefficient: −0.056). This interaction was confirmed by cross‐impulse response analysis, demonstrating that the effect of Inf B on *M. pneumoniae* persisted until lag 2. This suggests that the influence of Inf B on *M. pneumoniae* is predominantly short‐term, with diminishing long‐term effects.

**FIGURE 6:**
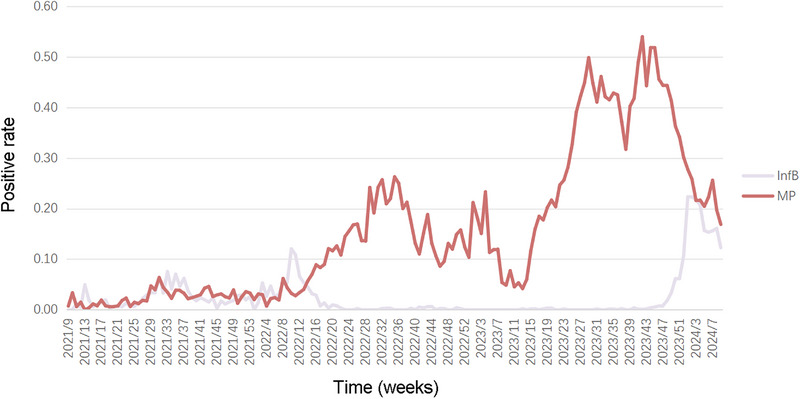
Weekly positive rates of influenza B virus and *Mycoplasma pneumonia*. Inf B, influenza B virus; MP, *Mycoplasma pneumoniae*.

Analysis of the Inf B virus and *M. pneumoniae* detection rates over three years revealed that *M. pneumoniae* outbreaks consistently followed the peak Inf B detection period (Figure 6). Following peak Inf B detection, a transient 3‐week decline in *M. pneumoniae* incidence was observed. This indicated that Inf B may precede or influence the epidemiological dynamics of *M. pneumoniae* outbreaks. The VAR model also identified subtle unstable interactions between HPIV and HMPV on HRSV (Table S4).

### Changes in detection and interactions of non‐bacterial pathogens during and after the pandemic

**FIGURE 7:**
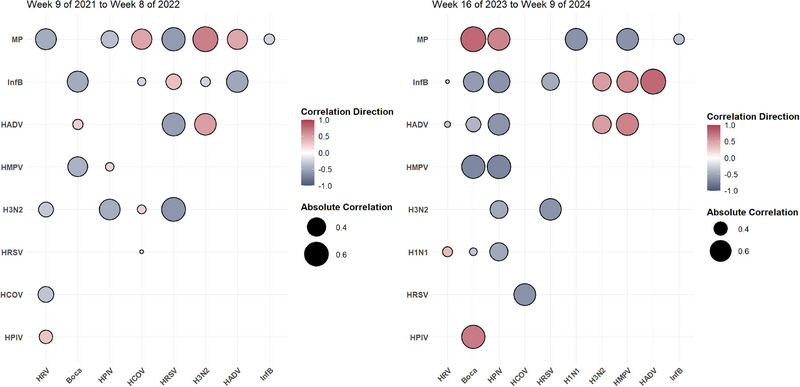
Correlation of respiratory non‐bacterial pathogens at the population level across two distinct phases. Blue–grey circles indicate negative correlations, red circles indicate positive correlations, and larger/darker circles represent stronger correlations. All correlations are statistically significant, as determined by Spearman's rank correlation (*P* < 0.05). Boca, bocavirus; Inf B, influenza B virus; HPIV, human parainfluenza virus; HRSV, human respiratory syncytial virus; HAdV, human adenovirus; HRV, human rhinovirus; HMPV, human metapneumovirus; HCOV, human coronavirus; MP, *Mycoplasma pneumoniae*.

Interactions between non‐bacterial pathogens during and after the pandemic were assessed using a comparative analysis of meaningful correlations (*P* < 0.001). During the pandemic, 24 correlations, including nine positive and 15 negative correlations, were detected (Figure 7). After the pandemic, 26 correlations (nine positive and 17 negative) were identified. Specifically, during and after the pandemic, a significant negative correlation was observed between *M. pneumoniae* and Inf B (−0.36 to −0.25) and between bocavirus and Inf B (−0.57 to −0.48). Conversely, a significant positive correlation was observed between H3N2 and HAdV (0.50 to 0.51). Additionally, significant negative correlations were observed between HMPV and bocavirus (−0.69 to −0.44), H3N2 and HPIV (−0.49 to −0.47), H3N2 and HRSV (−0.62 to −0.61), and HRSV and HCOV (−0.63 to −0.22). Following the pandemic, interactions among these pathogens increased, with Inf B interacting with up to eight others.

**FIGURE 8:**
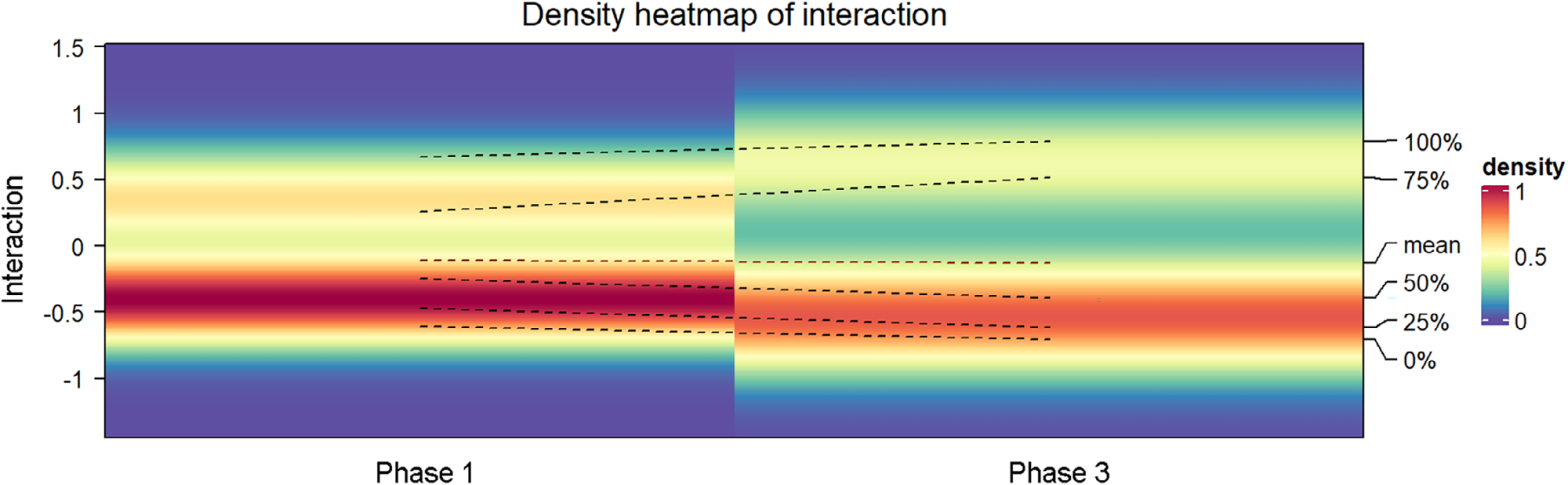
Temporal density plot of non‐bacterial pathogen detection correlation across various periods. Left y‐axis: annual Spearman correlation coefficients; percentages on the right indicate the mean, median, quartiles, and range of the correlation coefficients; six dashed lines depict annual variations; colour gradients on the right represent the density values of the distribution, with darker hues indicating higher density.

An in‐depth analysis was also performed on the correlations among pathogens over three years. A biphasic distribution of correlation coefficients was observed, characterized by peaks at approximately +0.5 and −0.5, with a higher prevalence of negative correlations. Notably, the average values of meaningful correlations (positive and negative) increased after the pandemic (Figure 8).

## DISCUSSION

In response to the emergence of SARS‐CoV‐2 with a high basic reproduction number, stringent NPIs have been introduced in Wenzhou and Ningbo. The initial pathogen detection rates remained suppressed immediately after restrictions were lifted, gradually returning to pre‐pandemic levels.^13^ Continued surveillance revealed that as NPIs were fully lifted, the infection rates of most non‐bacterial pathogens gradually returned to baseline, a trend consistent with global reports.^14^, ^15^, ^16^ This can be primarily attributed to the removal of NPIs, underscoring its influence in modifying health‐seeking behaviours, curtailing large‐scale gatherings, and mitigating the spread of respiratory infections. However, the cessation of NPIs has led to an increased incidence of respiratory infections. Although cessation of NPIs led to increased respiratory infection, detection rates of common non‐bacterial pathogens did not rise immediately and later declined.^17^ Notably, the detection rate of Inf A increased, consistent with other studies suggesting that exposure of susceptible populations, inadequate vaccine coverage, and interactions with SARS‐CoV‐2 may contribute to this trend.^18^, ^19^ These findings emphasize the potential synergistic effect among viruses and possible interaction with SARS‐CoV‐2.^20^, ^21^

In contrast to most respiratory viruses, HRV transmission has persisted despite the implementation of mitigation strategies during the COVID‐19 pandemic. HRV triggers an interferon response that blocks SARS‐CoV‐2 replication. Mathematical simulations suggested that this virus–virus interaction may impact the entire population.^22^ Meanwhile, HRV detection was lower in the subsequent stages of restriction easing than in the outbreak control period. Conversely, in New Zealand, HRV incidence increased during the later stages of the SARS‐CoV‐2 outbreak.^23^ This may be attributable to the high prevalence of Inf A, which, through interferon‐mediated mechanisms, limits HRV prevalence.^24^

Mixed infections involving HRV were more prevalent during and after the pandemic, which, in addition to the year‐round presence of HRV in the southeastern coastal region, may also be attributed to the relatively weaker exclusionary force of HRV against other viruses.^12^, ^25^ However, discrepancies between previous and current studies may stem from differences in host populations, methodologies, viral interactions, immunity in the context of SARS‐CoV‐2, and other undetermined viral interactions. Notably, during the later stages of the pandemic, double and triple co‐detections in the form of multiple combinations of the same pathogens increased, a trend observed internationally.^26^ This trend may be partly driven by the surge in *M. pneumoniae* infections.^27^ Additionally, it may be linked to the concept of ‘immune debt’, which refers to the absence of adaptive immunity in a population, potentially leading to the outbreak of various respiratory diseases.^28^

HRSV and HMPV exhibited significant delays in seasonal infection peaks. This may be attributed to the protective effects of mitigation measures, including masks, hand hygiene, and physical distancing, which likely reduced transmission during the early stages of lockdown relaxation.^23^ In particular, the lowest transmission levels of HMPV in older children occurred during the pandemic when school closures were prevalent and resurged with the partial reopening of daycares and schools.^11^, ^29^

HPIV, characterized by slow replication and a prolonged infection cycle, facilitate greater coexistence with other respiratory viruses, enhancing the likelihood of complex viral interactions.^30^, ^31^ Empirical evidence has demonstrated a positive correlation between human coronaviruses and HPIV in pediatric populations in metropolitan areas, such as Shanghai, Beijing, and Guangzhou.^32^, ^33^, ^34^ However, we detected a negative correlation between HPIV and influenza H3N2. This is consistent with studies in the Netherlands and China, which documented comparative peak shift patterns between HPIV and influenza over several years and differing trends within the same season, highlighting the influence of HPIV and influenza H3N2.^35^, ^36^, ^37^

Previous studies posited that certain mechanisms may be responsible for the occurrence of mixed infections with specific viral pairs. Horemheb‐Rubio et al.^38^ documented a coinfection exclusion score between HPIV‐4 and H3N2, as well as between HPIV‐1 and human coronavirus NL63, indicating a statistically significant correlation between the test outcomes for these pairs, suggesting a heightened likelihood of interaction. We observed that viral co‐infections were less prevalent than anticipated within a month of the peak incidence of each virus, suggesting that the biological mechanisms of viral exclusion or host response are of considerable importance.^39^ Given the high frequency of mixed infections, particularly among pediatric populations. These interactions likely occur frequently through host immune responses, changes in host susceptibility, or direct viral interactions.^40^, ^41^, ^42^

Numerous studies have identified interactions between SARS‐CoV‐2 and other viruses. Patients with COVID‐19 are often co‐infected with either Inf A or B, depending on the predominant influenza type at a given time.^43^ For example, in mid‐January 2020, patients with COVID‐19 who were hospitalized in Wuhan were frequently co‐infected with Inf B. However, after January 28, 2020, Inf A became the prevalent co‐pathogen.^44^ Additionally, a decline has been reported in the rate of mixed infections during the COVID‐19 control period compared to the acute outbreak period, followed by a rapid return to previous levels of mixed infections after restrictions.^45^ Further analyses are needed to determine whether the biological mechanisms underlying SARS‐CoV‐2 interference with other non‐bacterial pathogens can explain the changes in co‐infections.

Our findings suggest that the interplay between viral and atypical pathogens influences epidemiological patterns of respiratory infections. Specifically, a negative interaction was consistently observed between Inf B and *M. pneumoniae*. Additionally, a decrease in the detection of *M. pneumoniae* was observed during *M. pneumoniae* outbreaks in Wenzhou and Ningbo, coinciding with brief outbreaks of Inf B. This phenomenon was particularly common in 2023 when a major outbreak of macrolide‐resistant *M. pneumoniae* occurred in central, northern, and eastern China^46^, ^47^, ^48^; conversely, the reduced detection of *M. pneumoniae* in previous studies did not have a clear sequential relationship with the brief outbreak of Inf B.^48^ Similarly, other researchers found a correlation between *M. pneumoniae* and Inf B during this period.^49^ Pathogen infection stimulates the innate immune system and the release of interferons that inhibit subsequent infections by other pathogens.^50^ Pathogens may compete for limited host resources. Additionally, infection with certain pathogens may inhibit the entry of other pathogens into the host cells.^51^

The higher incidence of co‐infections during winter aligns with the typical seasonal spread of multiple respiratory pathogens, but distinct summer peaks were also observed.^52^ Research indicates a consistent interaction between respiratory viruses at the human population level, independent of seasonal variations.^53^ However, our findings suggest that the nature of these viral interactions was altered after the pandemic. The emergence and widespread transmission of SARS‐CoV‐2 may have influenced its interactions with other respiratory viruses.

This study had some limitations. First, it focuses exclusively on hospitalized children. Thus, the findings must be validated in outpatient or community settings, leading to bias in the assessment of pathogen prevalence burden. Second, the duration of the study spanned three years, necessitating further verification over a longer temporal range. Additionally, our study lacked data on SARS‐CoV‐2 detection, necessitating a reference to the existing literature for related conclusions. However, this approach may still fail to account for SARS‐CoV‐2 co‐infections with other pathogens or its direct inhibitory effects on the prevalence of other pathogens. Another limitation was the lack of data regarding antiviral treatment administered to patients prior to sample collection. Finally, our study was conducted along the eastern coast of China. Additional data from other geographical regions are necessary to comprehensively evaluate the characteristics of viral interactions.

In summary, this study used a large‐scale dataset of multi‐pathogen detection to characterize the interactions among respiratory pathogens and the impact of the COVID‐19 pandemic on these interactions. This pandemic and its associated prevention and control measures have profoundly influenced respiratory infections. Therefore, continued surveillance of the epidemiology of various respiratory pathogens remains crucial, particularly as NPIs have become less necessary. Future intervention strategies, including the formulation of combination vaccines, should consider the spatial and temporal interactions among pathogens to enhance the efficacy of controlling their transmission.

## CONFLICT OF INTEREST

The authors declare no conflict of interest.

## Supporting information



Supporting Information

Supporting Information
