# Supplementary material for: Interaction analysis of non‐bacterial respiratory pathogens during and after the coronavirus disease 2019 pandemic in two cities along the eastern coast of China
Source: Pediatr Investig. 2026 Jan 19;10(1):47–59. doi: 10.1002/ped4.70034 (PMC12921633; doi:10.1002/ped4.70034)

Fig S1 Pathogen Correlation: HRV vs Boca. Scatter plot showing the correlation between the weekly detection rates of HRV and Boca virus. The Spearman correlation coefficient is 0.262 with a p-value of 0.000852, indicating a positive correlation between the two pathogens.

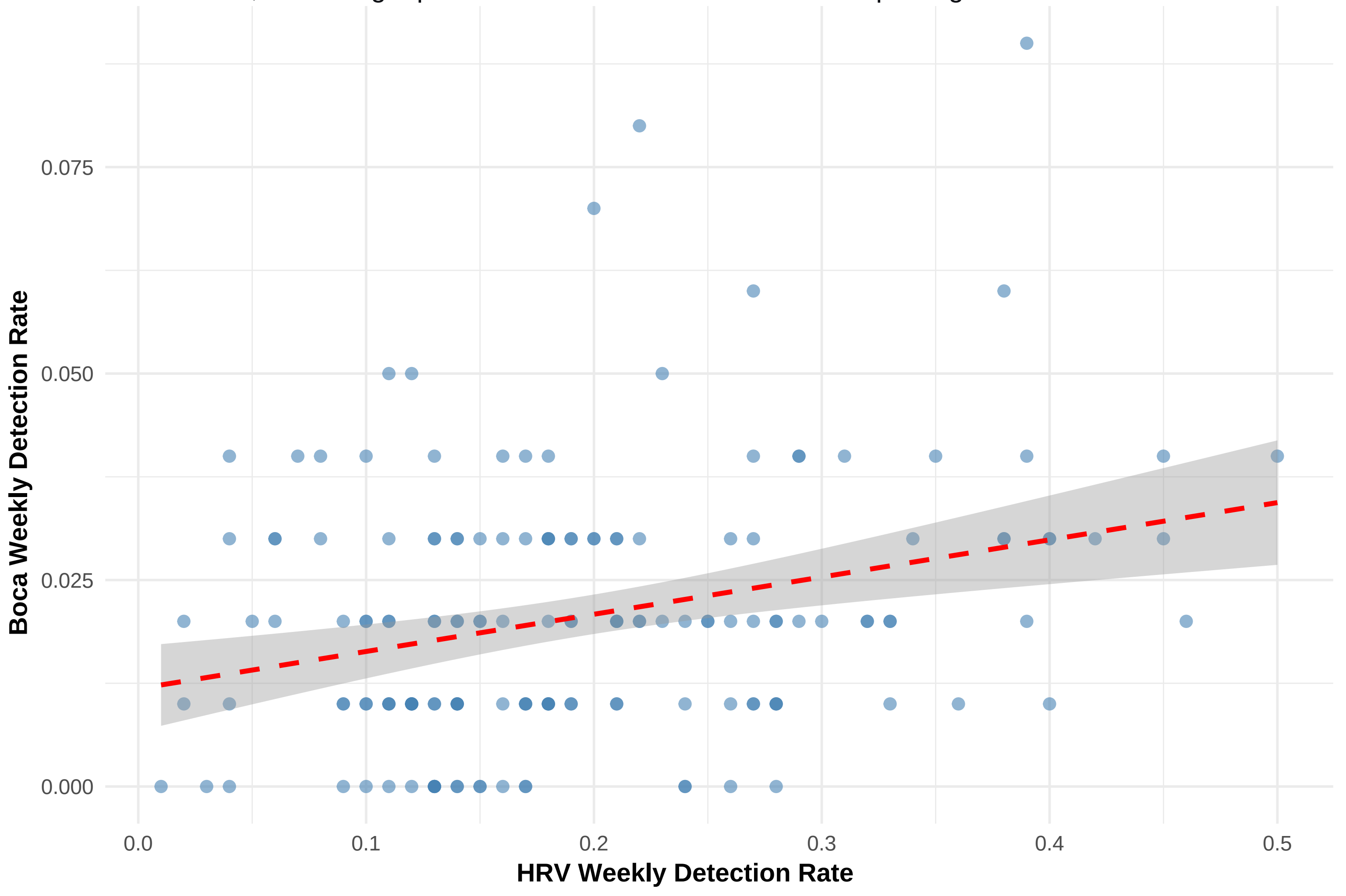

Fig S2 Pathogen Correlation: HRV vs HPI. Scatter plot showing the correlation between the weekly detection rates of HRV and HPIV. The Spearman correlation coefficient is 0.429 with a p-value of 6.79e-08, indicating a positive correlation between the two pathogens.

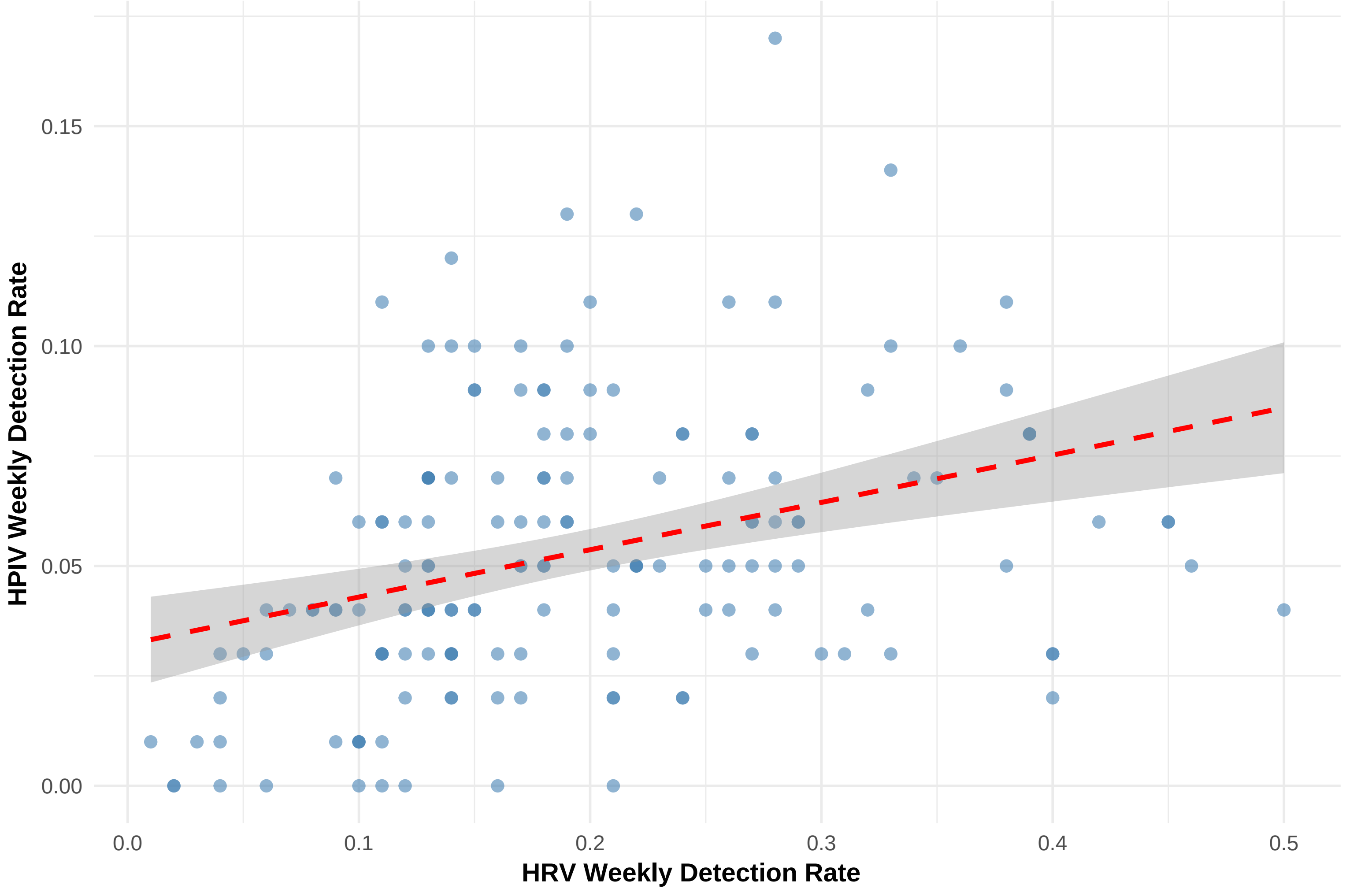

Fig S3 Pathogen Correlation: HRV vs H3N2. Scatter plot showing the correlation between the weekly detection rates of HRV and H3N2. The Spearman correlation coefficient is -0.227 with a p - value of 0.00291, indicating a negative correlation between the two pathogens.

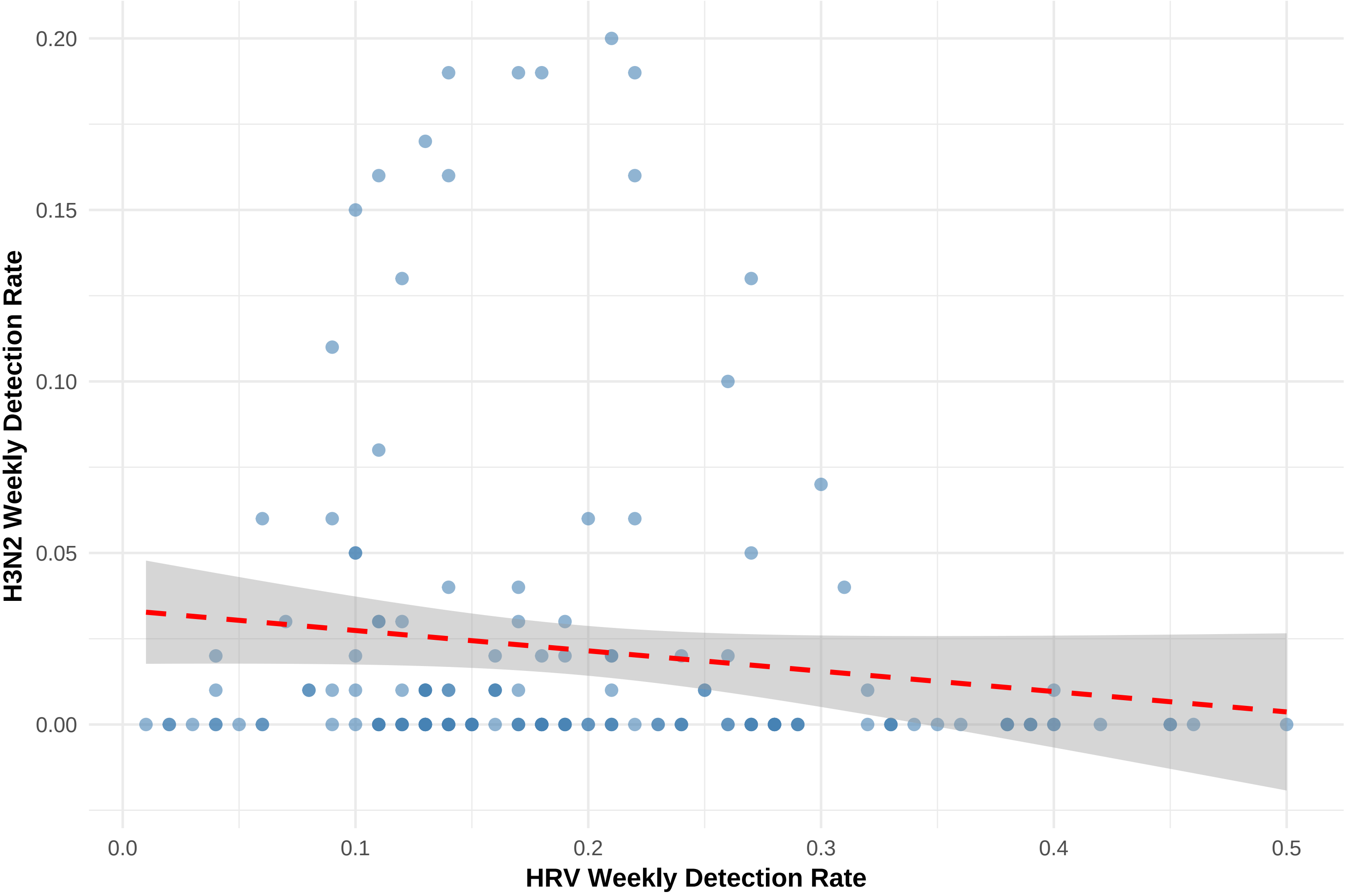

Fig S4 Pathogen Correlation: HRV vs HADV. Scatter plot showing the correlation between the weekly detection rates of HRV and HADV. The Spearman correlation coefficient is -0.123 with a p - value of 0.00423, indicating a negative correlation between the two pathogens.

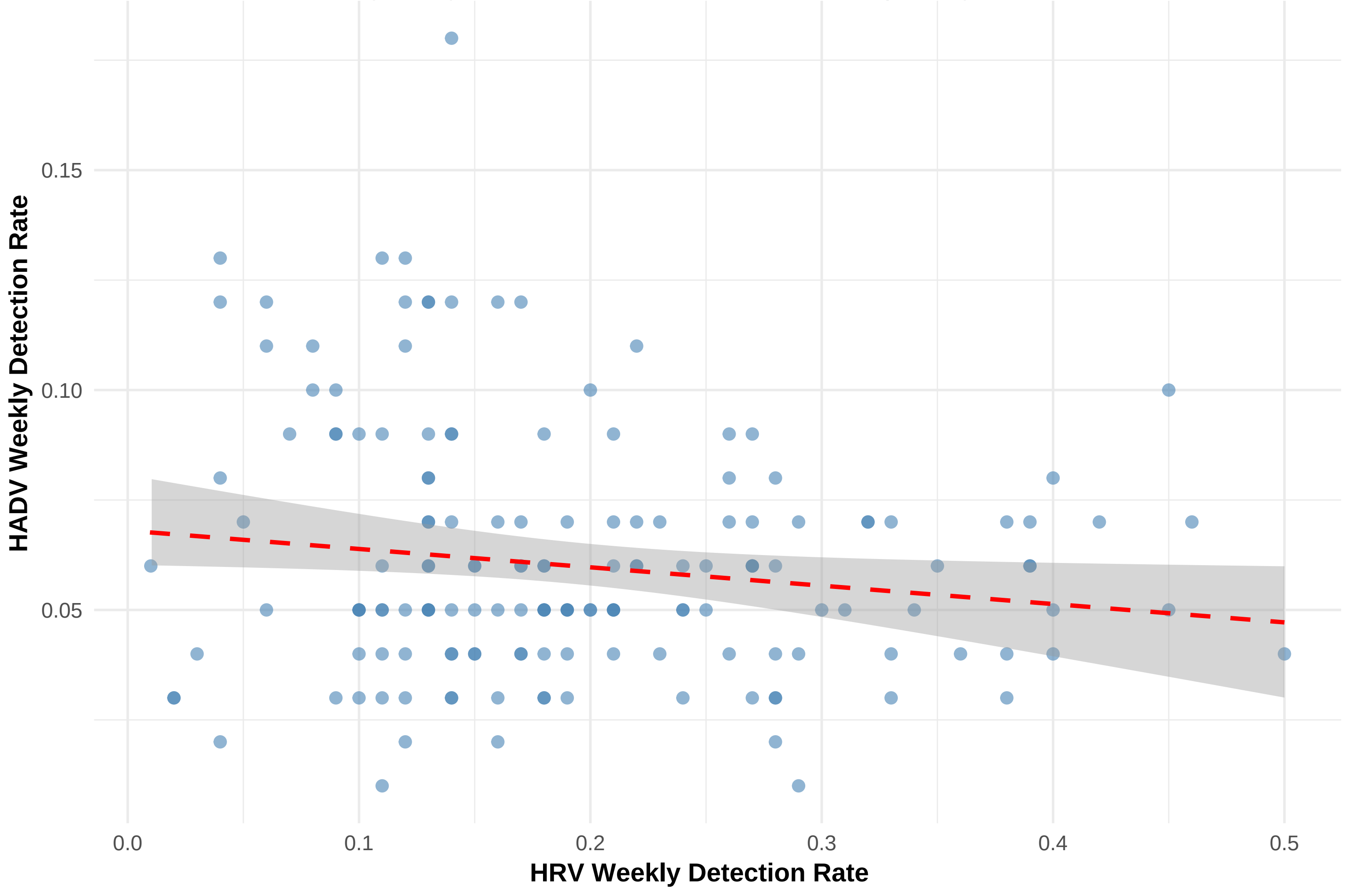

Fig S5 Pathogen Correlation: HRV vs MP. Scatter plot showing the correlation between the weekly detection rates of HRV and MP. The Spearman correlation coefficient is -0.397 with a p-value of 2.19e-07, indicating a negative correlation between the two pathogens.

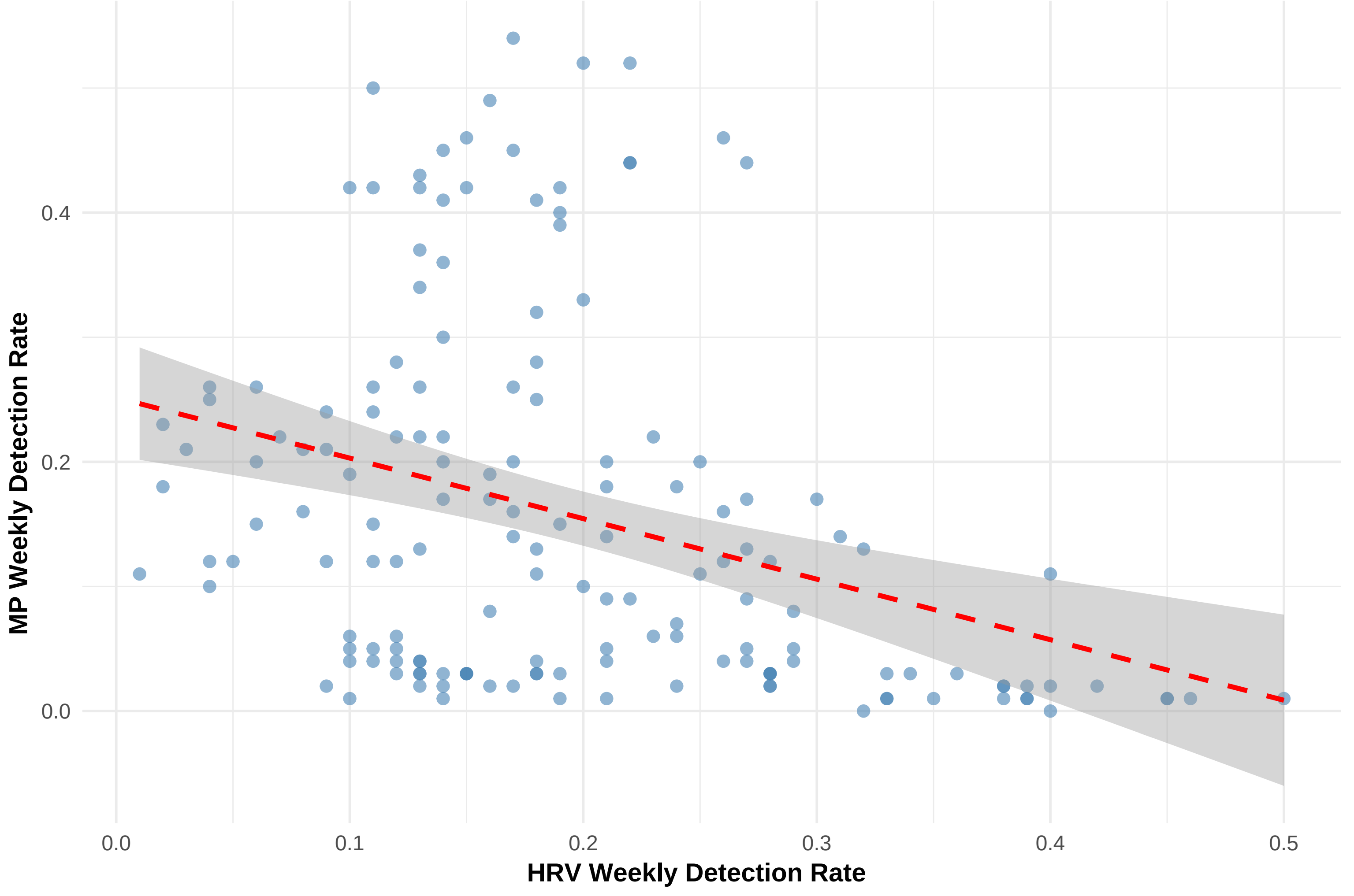

Fig S6 Pathogen Correlation: Boca vs HPIV. Scatter plot showing the correlation between the weekly detection rates of Boca virus and HPIV. The Spearman correlation coefficient is 0.355 with a p-value of 4.27e-06, indicating a positive correlation between the two pathogens.

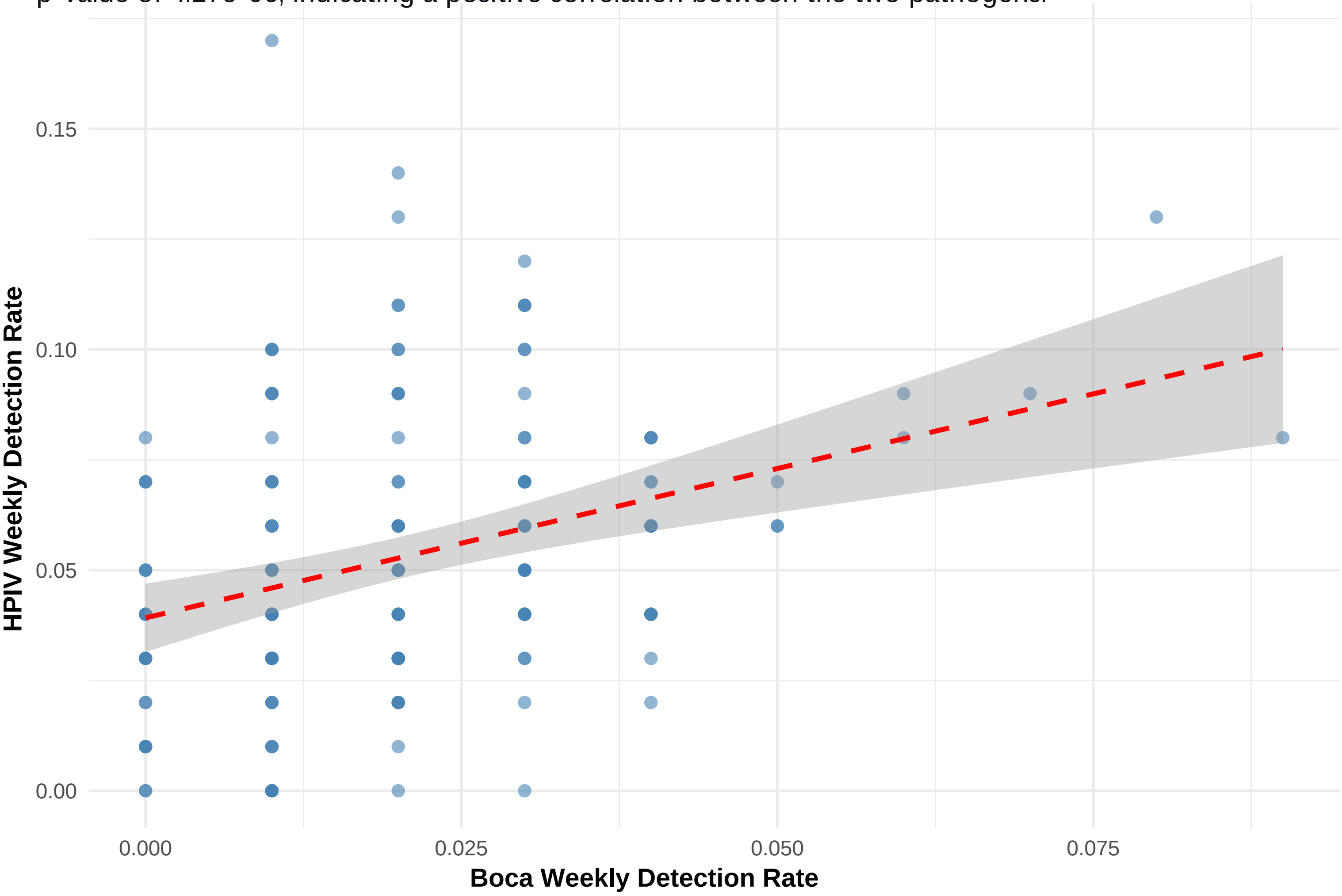

Fig S7 Pathogen Correlation: Boca vs H1N1. Scatter plot showing the correlation between the weekly detection rates of Boca virus and H1N1. The Spearman correlation coefficient is -0.277 with a p-value of 0.000413, indicating a negative correlation between the two pathogens.

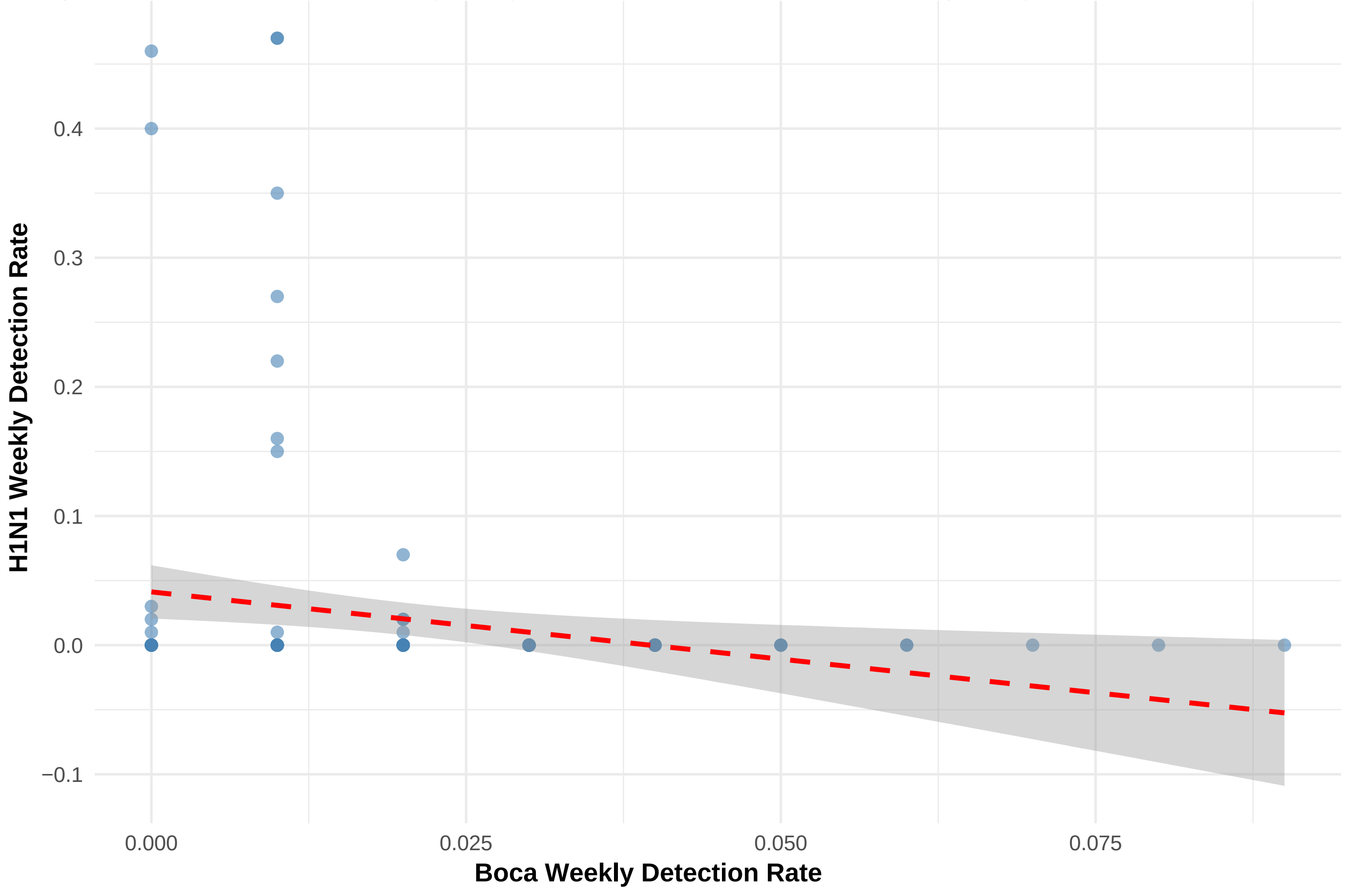

Fig S8 Pathogen Correlation: Boca vs HMPV. Scatter plot showing the correlation between the weekly detection rates of Boca virus and HMPV. The Spearman correlation coefficient is -0.343 with a p-value of 9.64e-06, indicating a negative correlation between the two pathogens.

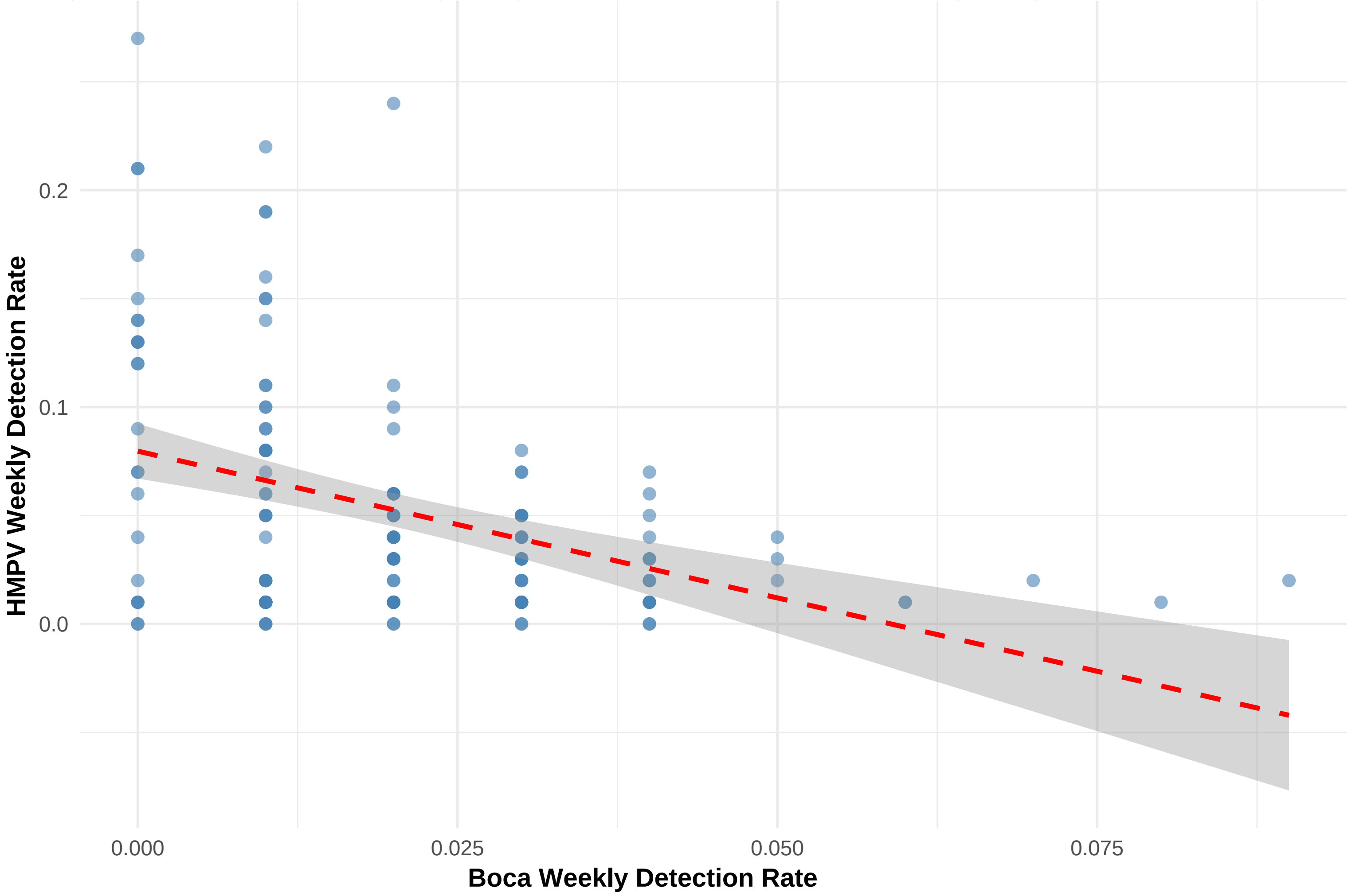

Fig S9 Pathogen Correlation: Boca vs InfB. Scatter plot showing the correlation between the weekly detection rates of Boca virus and Influenza B. The Spearman correlation coefficient is -0.265 with a p-value of 0.000742, indicating a negative correlation between the two pathogens.

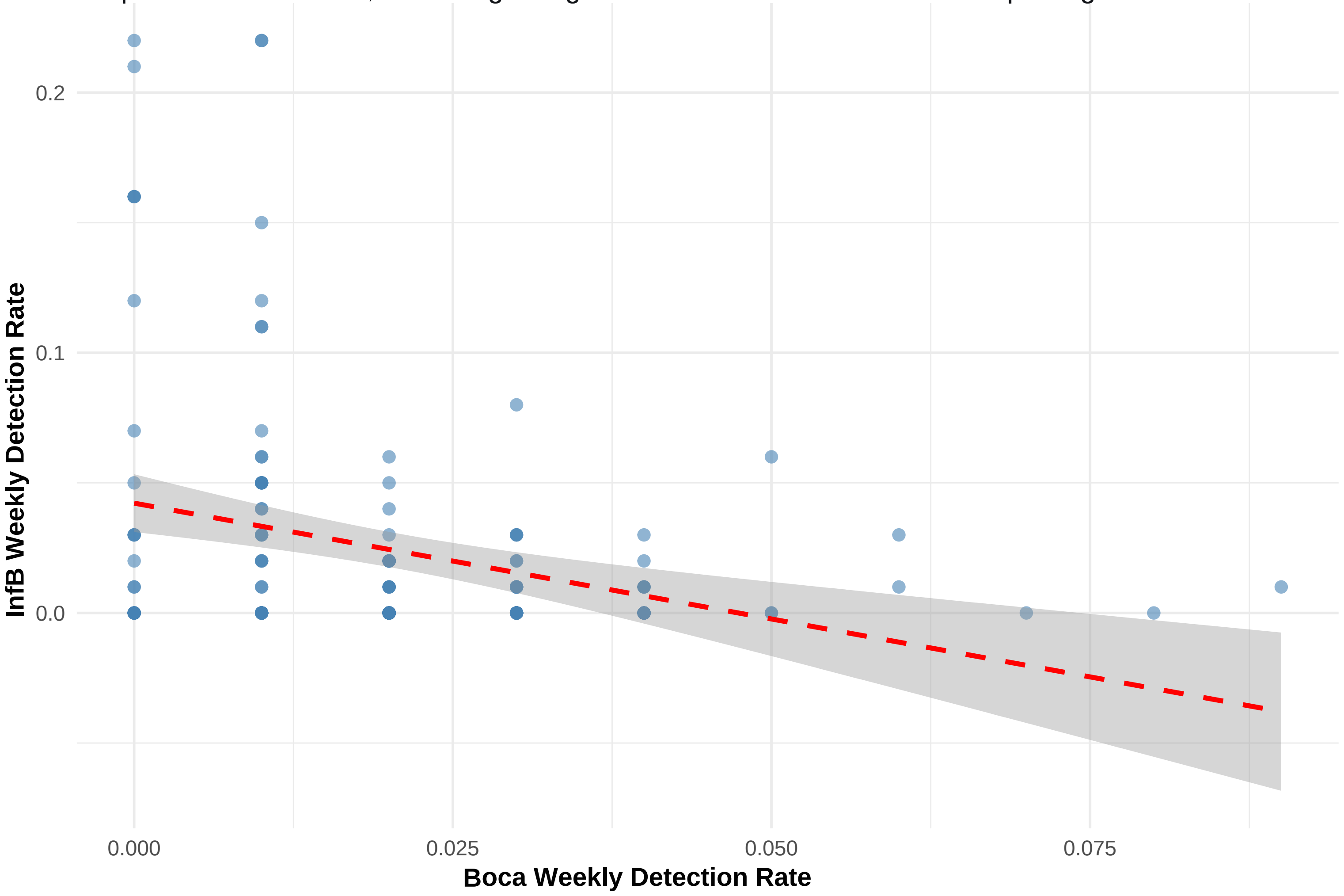

Fig S10 Pathogen Correlation: HPIV vs HCOV. Scatter plot showing the correlation between the weekly detection rates of HPIV and HCOV. The Spearman correlation coefficient is 0.256 with a p - value of 0.00111, indicating a positive correlation between the two pathogens.

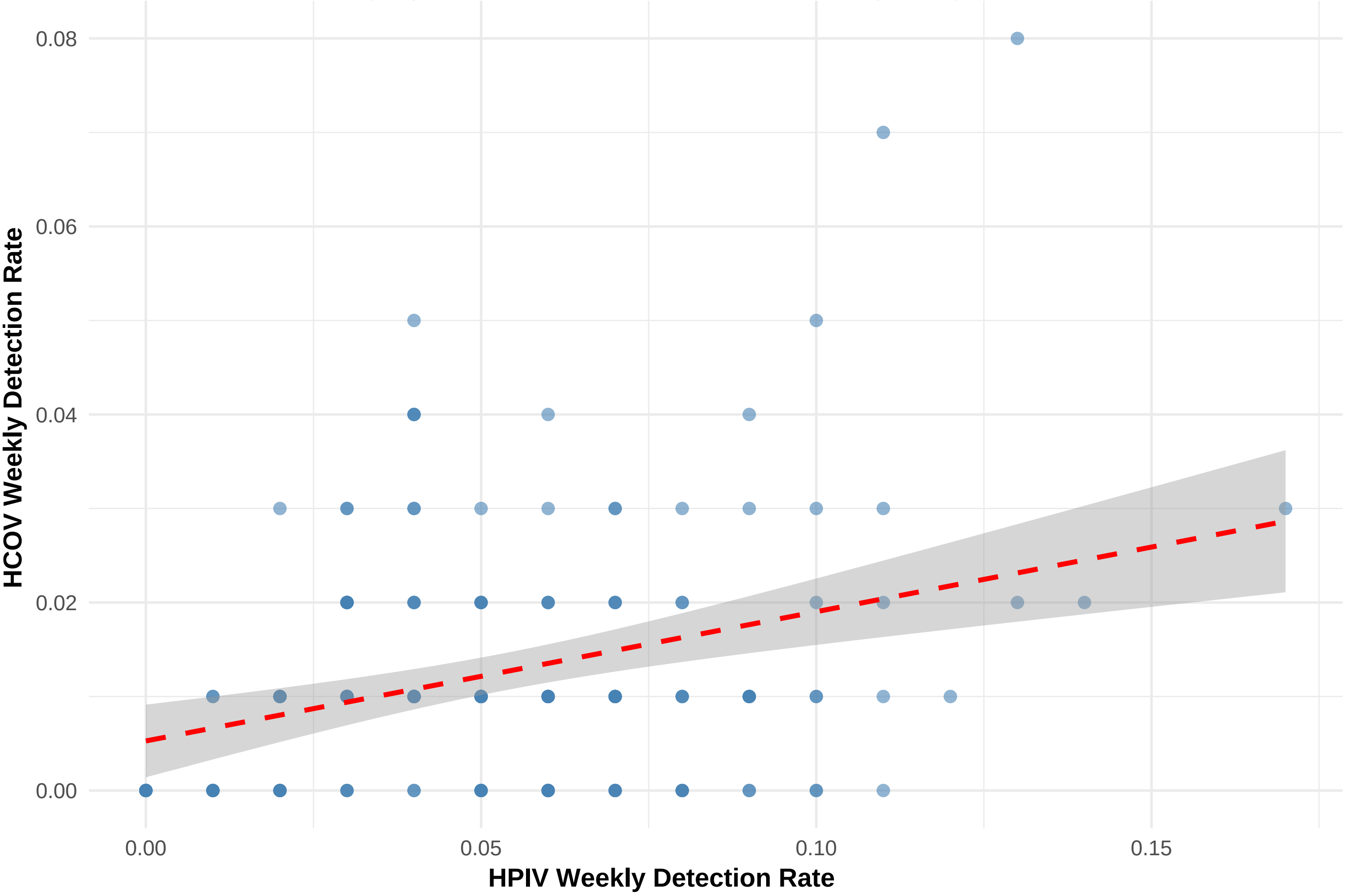

Fig S11 Pathogen Correlation: HPIV vs HRSV. Scatter plot showing the correlation between the weekly detection rates of HPIV and HRSV. The Spearman correlation coefficient is 0.312 with a p - value of 6.19e-05, indicating a positive correlation between the two pathogens.

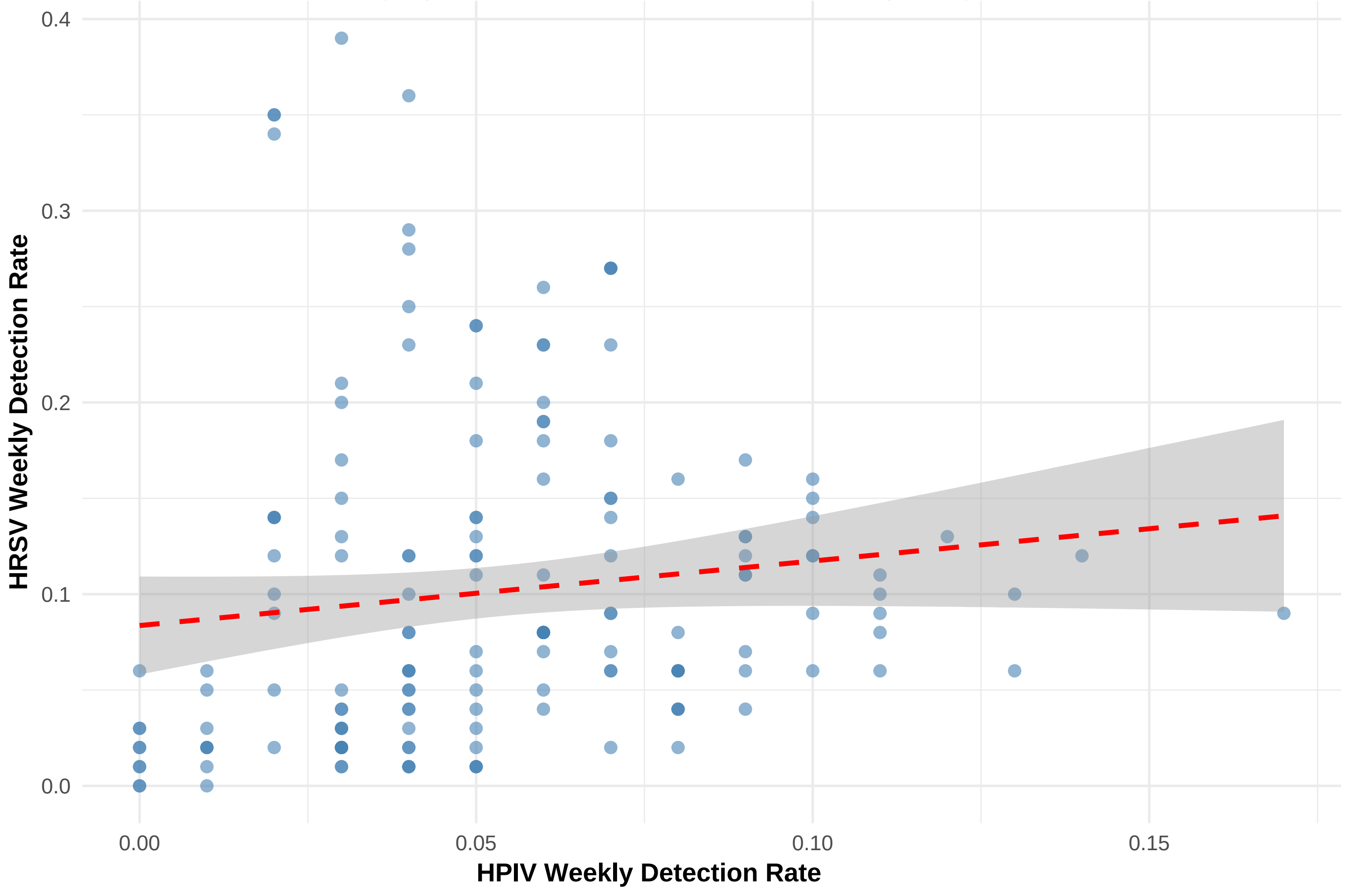

Fig S12 Pathogen Correlation: HPIV vs H1N1. Scatter plot showing the correlation between the weekly detection rates of HPIV and H1N1. The Spearman correlation coefficient is -0.43 with a p-value of 1.52e-08, indicating a negative correlation between the two pathogens.

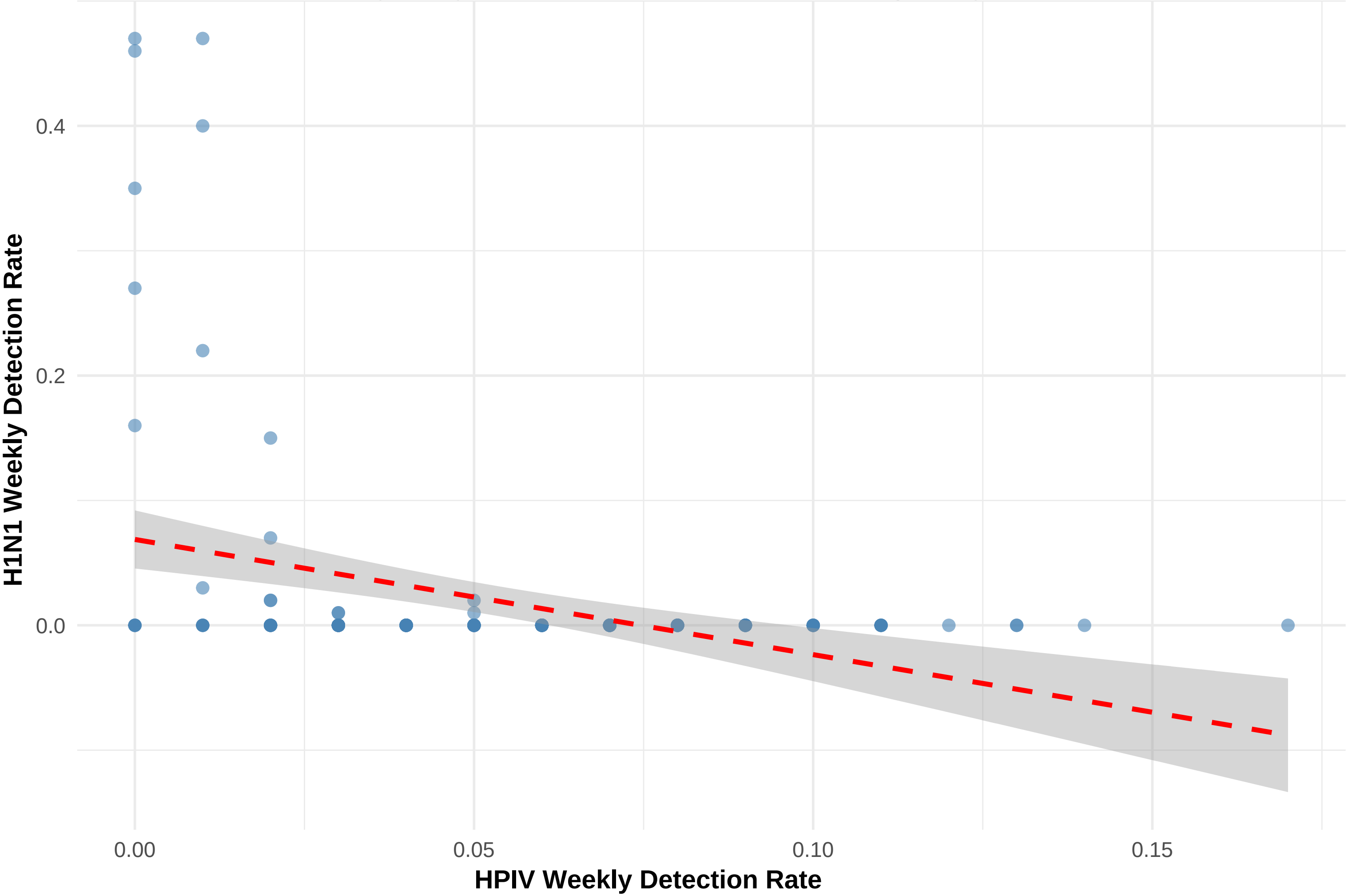

Fig S13 Pathogen Correlation: HPIV vs H3N2. Scatter plot showing the correlation between the weekly detection rates of HPIV and H3N2. The Spearman correlation coefficient is -0.434 with a p - value of 1.1e-08, indicating a negative correlation between the two pathogens.

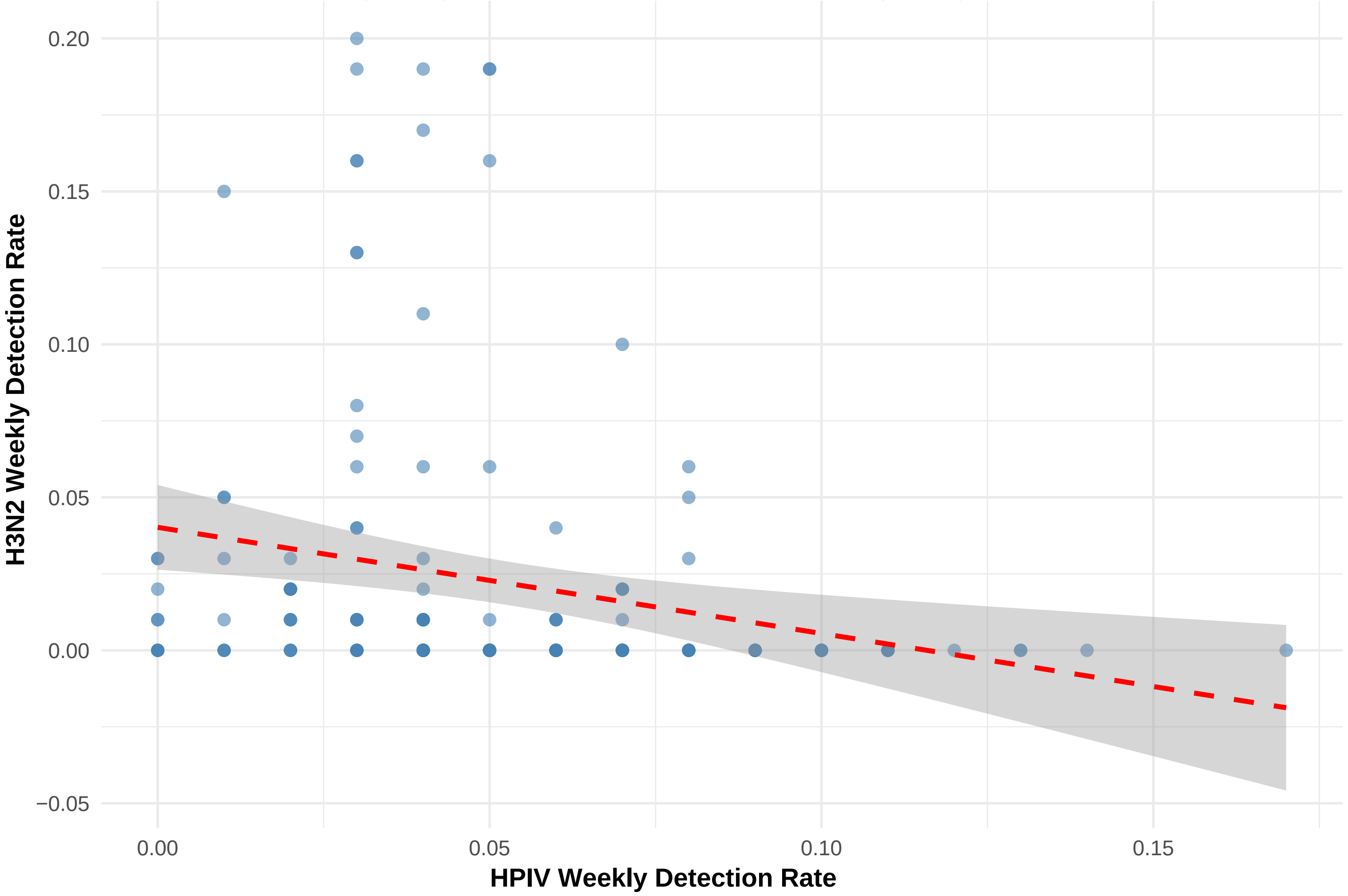

Fig S14 Pathogen Correlation: HPIV vs HADV. Scatter plot showing the correlation between the weekly detection rates of HPIV and HADV. The Spearman correlation coefficient is -0.161 with a p-value of 0.038, indicating a negative correlation between the two pathogens.

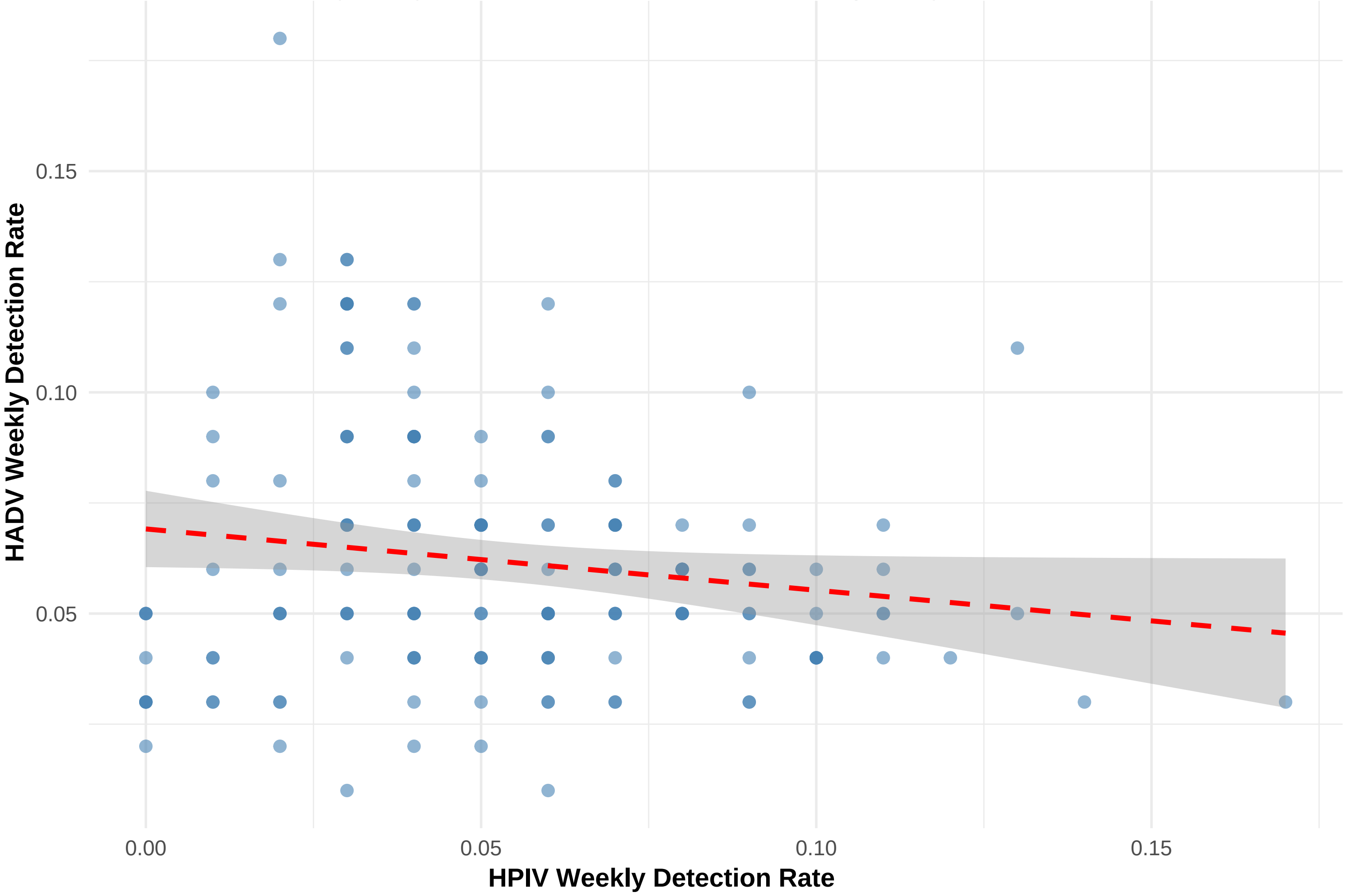

Fig S15 Pathogen Correlation: HCOV vs HRSV. Scatter plot showing the correlation between the weekly detection rates of HCOV and HRSV. The Spearman correlation coefficient is -0.166 with a p-value of 0.0368, indicating a negative correlation between the two pathogens.

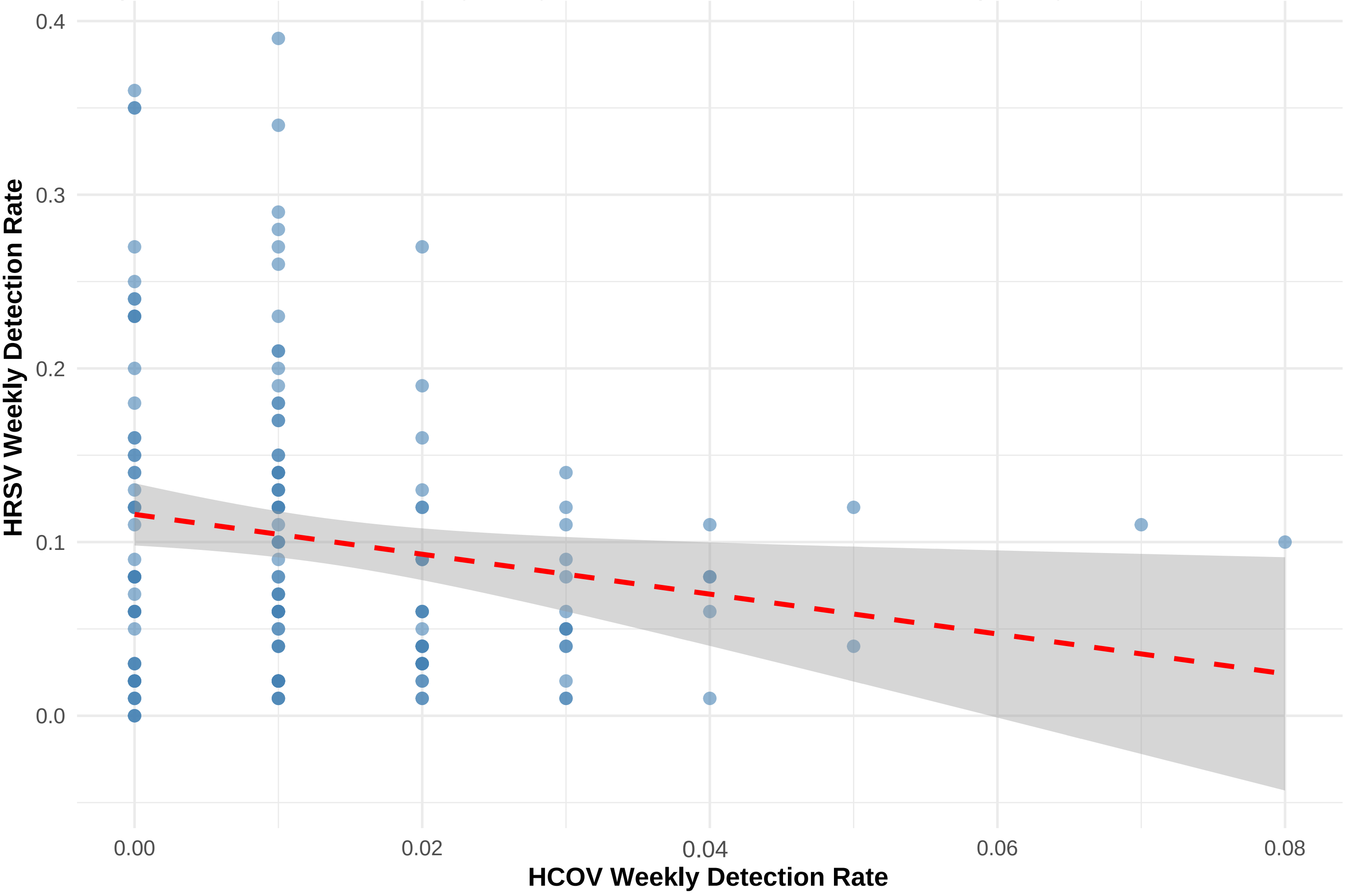

Fig S16 Pathogen Correlation: HCOV vs H1N1. Scatter plot showing the correlation between the weekly detection rates of HCOV and H1N1. The Spearman correlation coefficient is -0.321 with a p-value of 3.61e-05, indicating a negative correlation between the two pathogens.

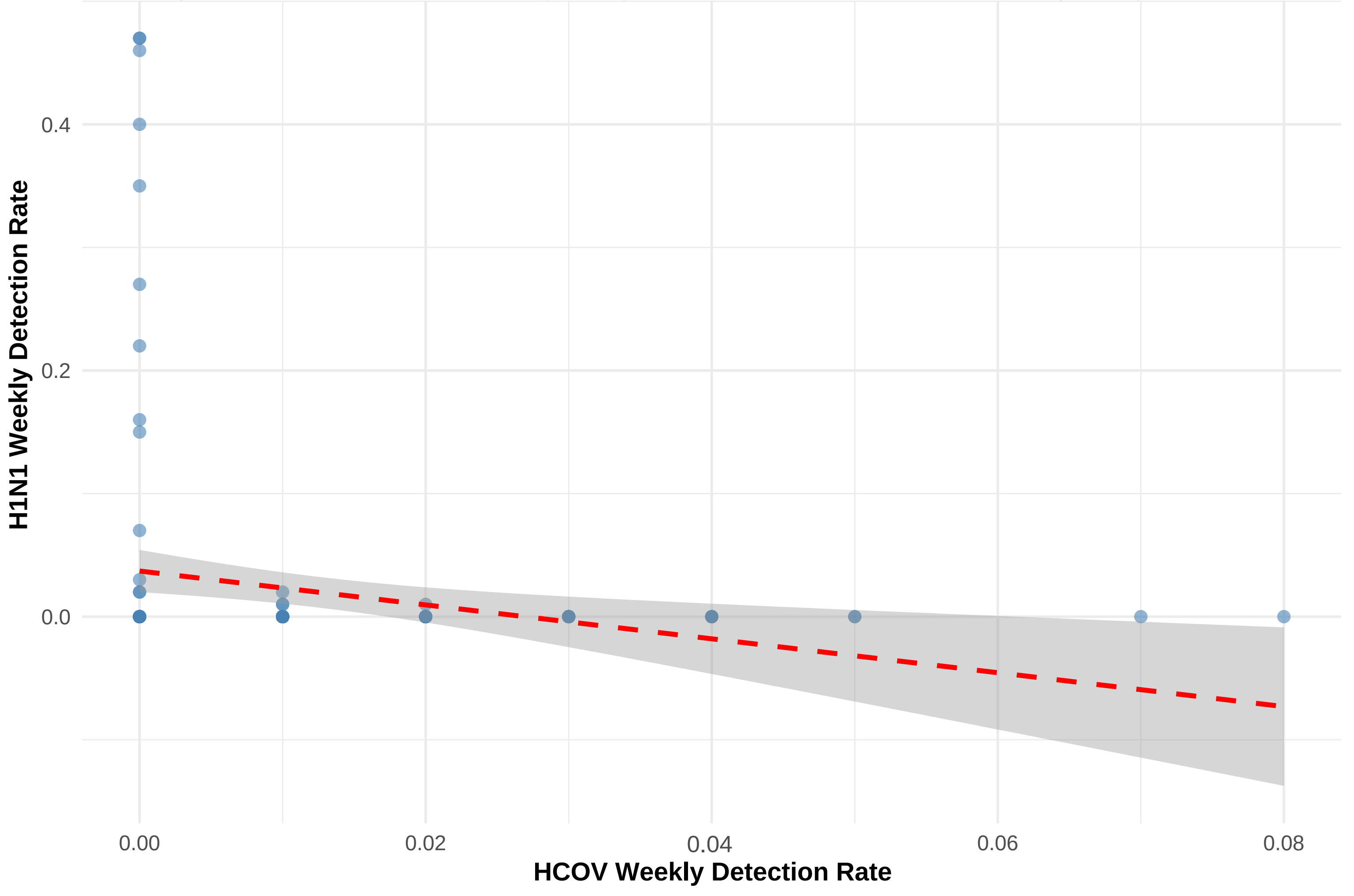

Fig S17 Pathogen Correlation: HCOV vs HADV. Scatter plot showing the correlation between the weekly detection rates of HCOV and HADV. The Spearman correlation coefficient is 0.221 with a p-value of 0.00514, indicating a positive correlation between the two pathogens.

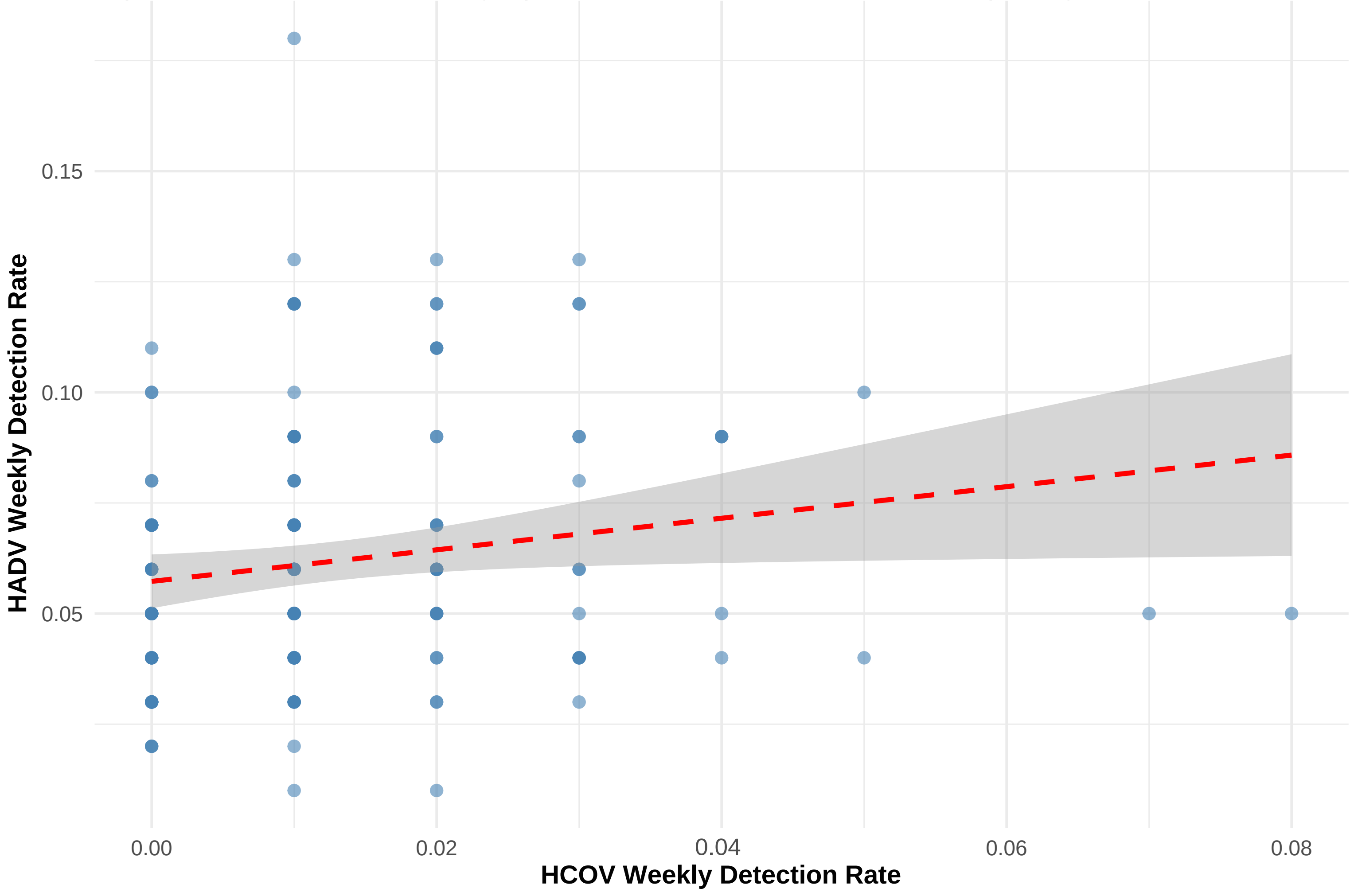

Fig S18 Pathogen Correlation: HCOV vs InfB. Scatter plot showing the correlation between the weekly detection rates of HCOV and Influenza B. The Spearman correlation coefficient is 0.150 with a p-value of 0.0456, indicating a positive correlation between the two pathogens.

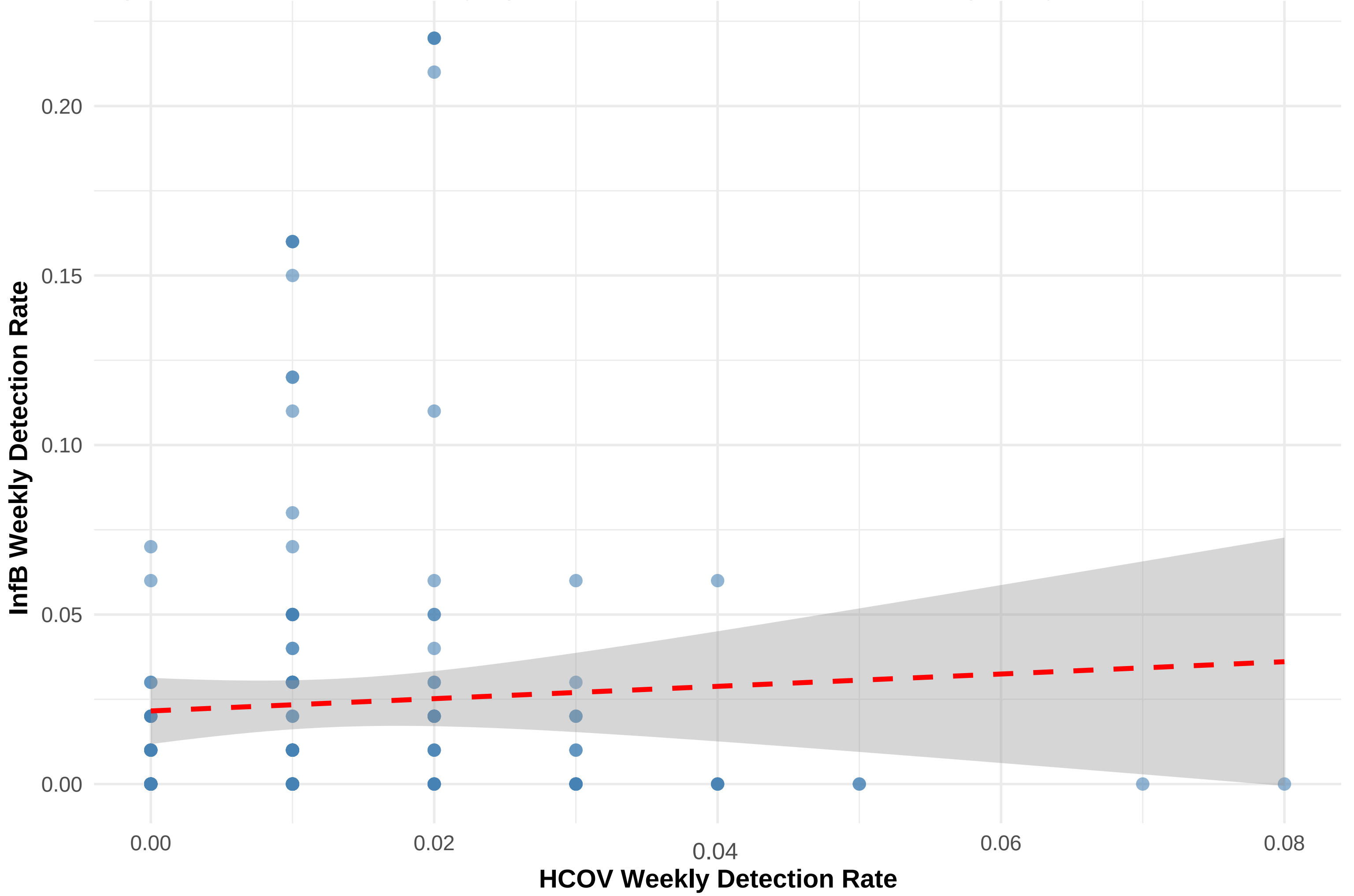

Fig S19 Pathogen Correlation: HCOV vs MP. Scatter plot showing the correlation between the weekly detection rates of HCOV and MP. The Spearman correlation coefficient is 0.267 with a p-value of 0.000657, indicating a positive correlation between the two pathogens.

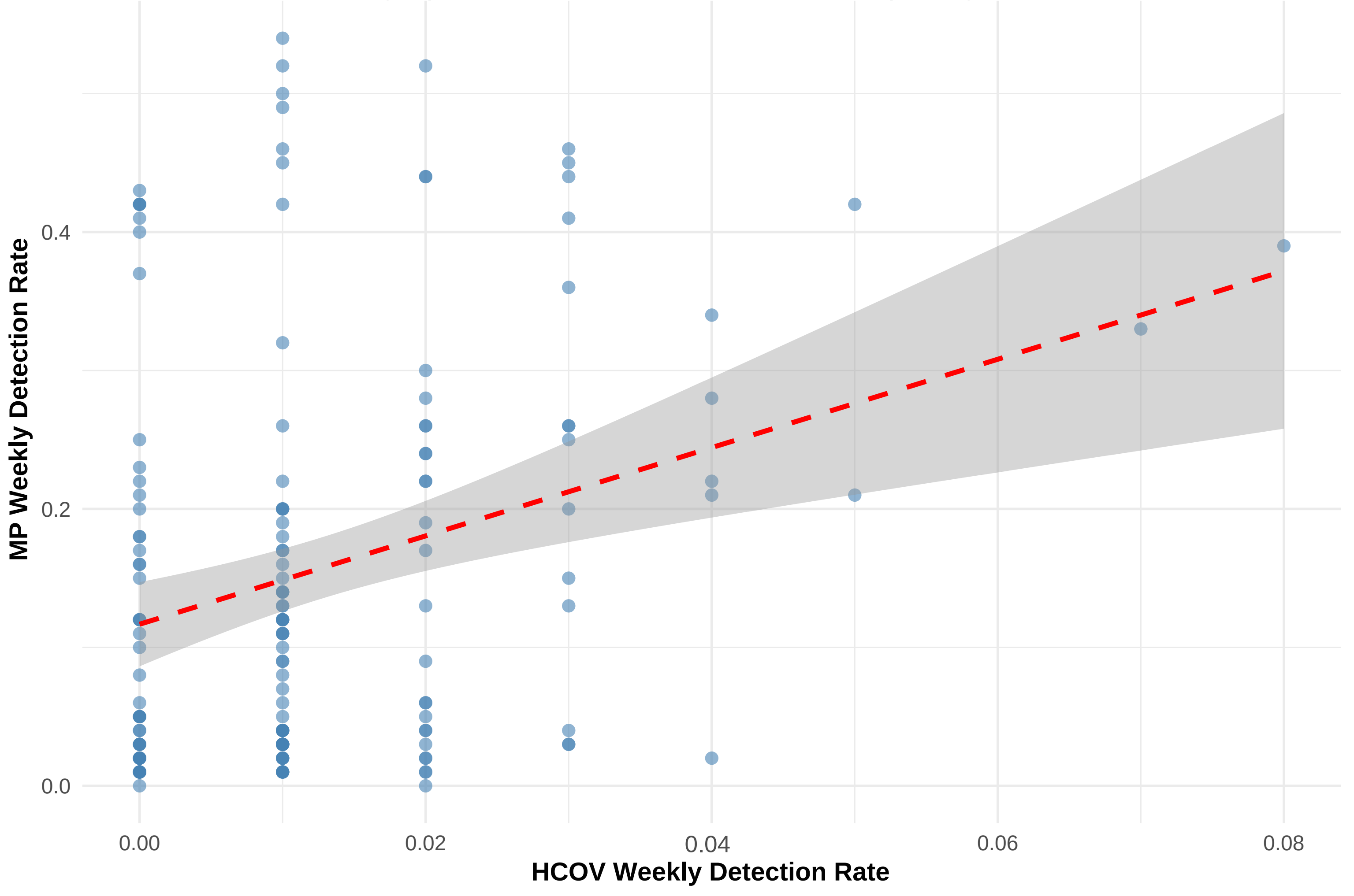

Fig S20 Pathogen Correlation: HRSV vs H3N2. Scatter plot showing the correlation between the weekly detection rates of HRSV and H3N2. The Spearman correlation coefficient is -0.471 with a p-value of 3.79e-10, indicating a negative correlation between the two pathogens.

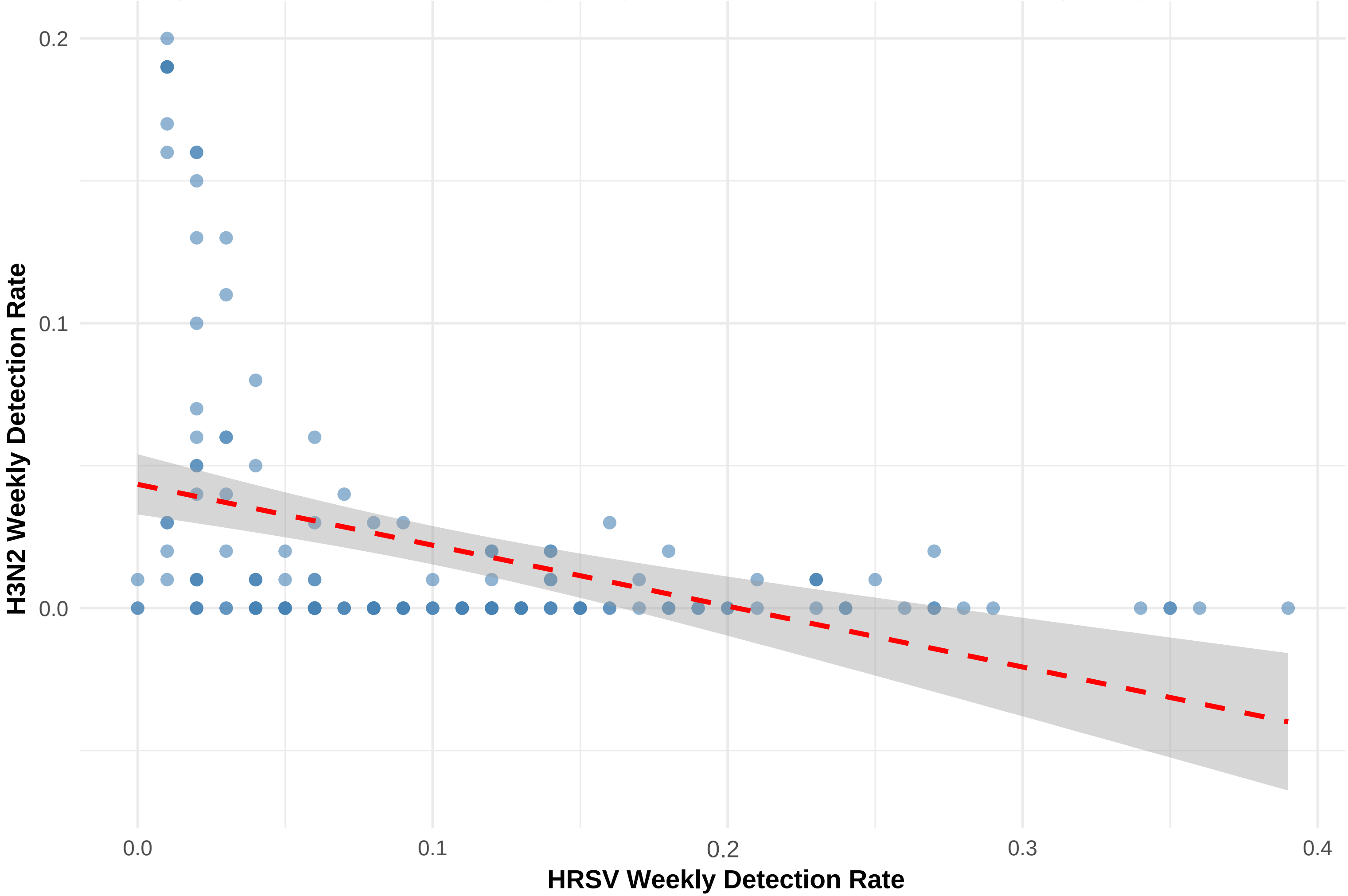

Fig S21 Pathogen Correlation: HRSV vs HADV. Scatter plot showing the correlation between the weekly detection rates of HRSV and HADV. The Spearman correlation coefficient is -0.239 with a p-value of 0.00244, indicating a negative correlation between the two pathogens.

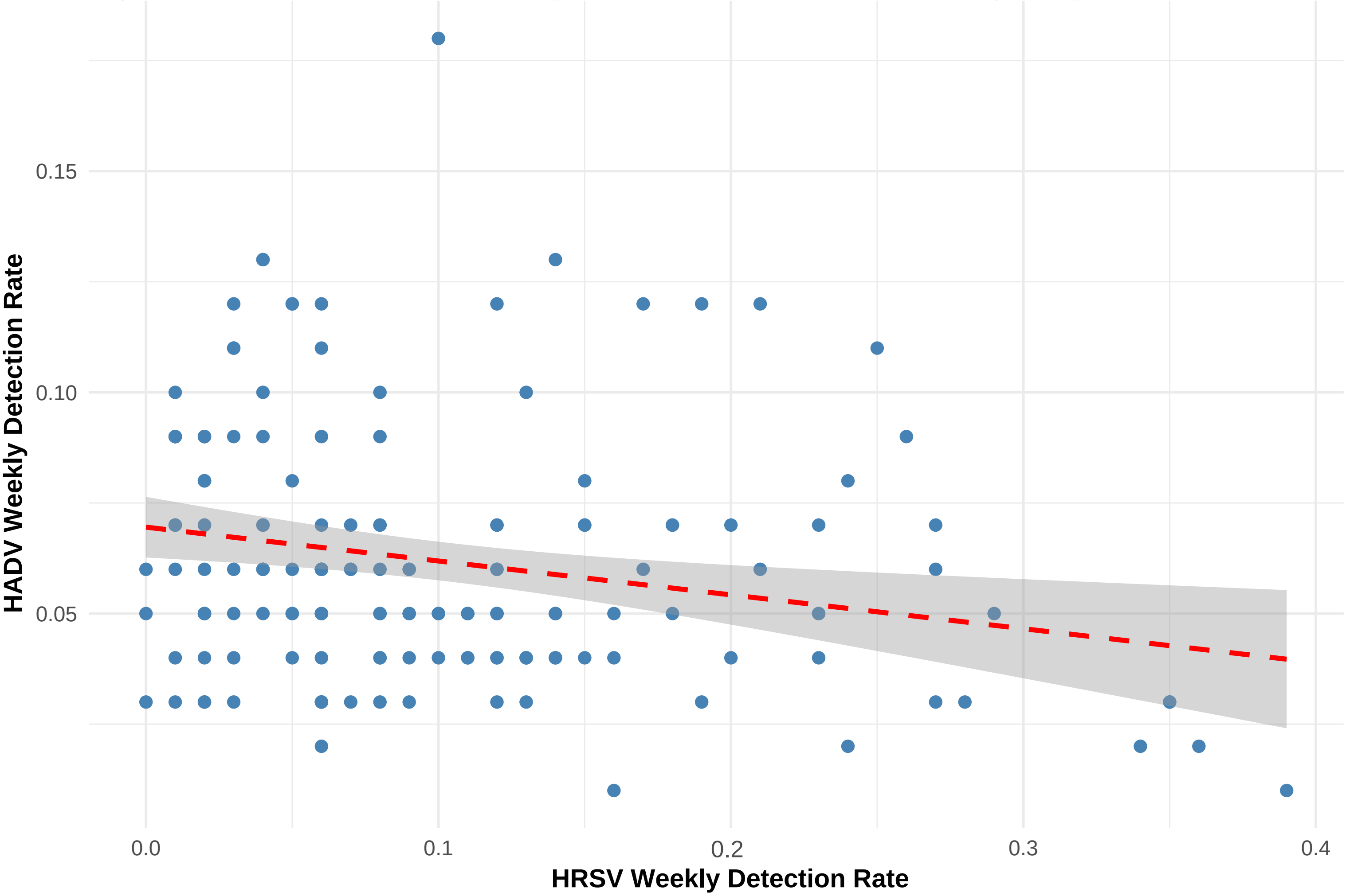

Fig S22 Pathogen Correlation: HRSV vs InfB. Scatter plot showing the correlation between the weekly detection rates of HRSV and Influenza B. The Spearman correlation coefficient is 0.165 with a p-value of 0.0376, indicating a positive correlation between the two pathogens.

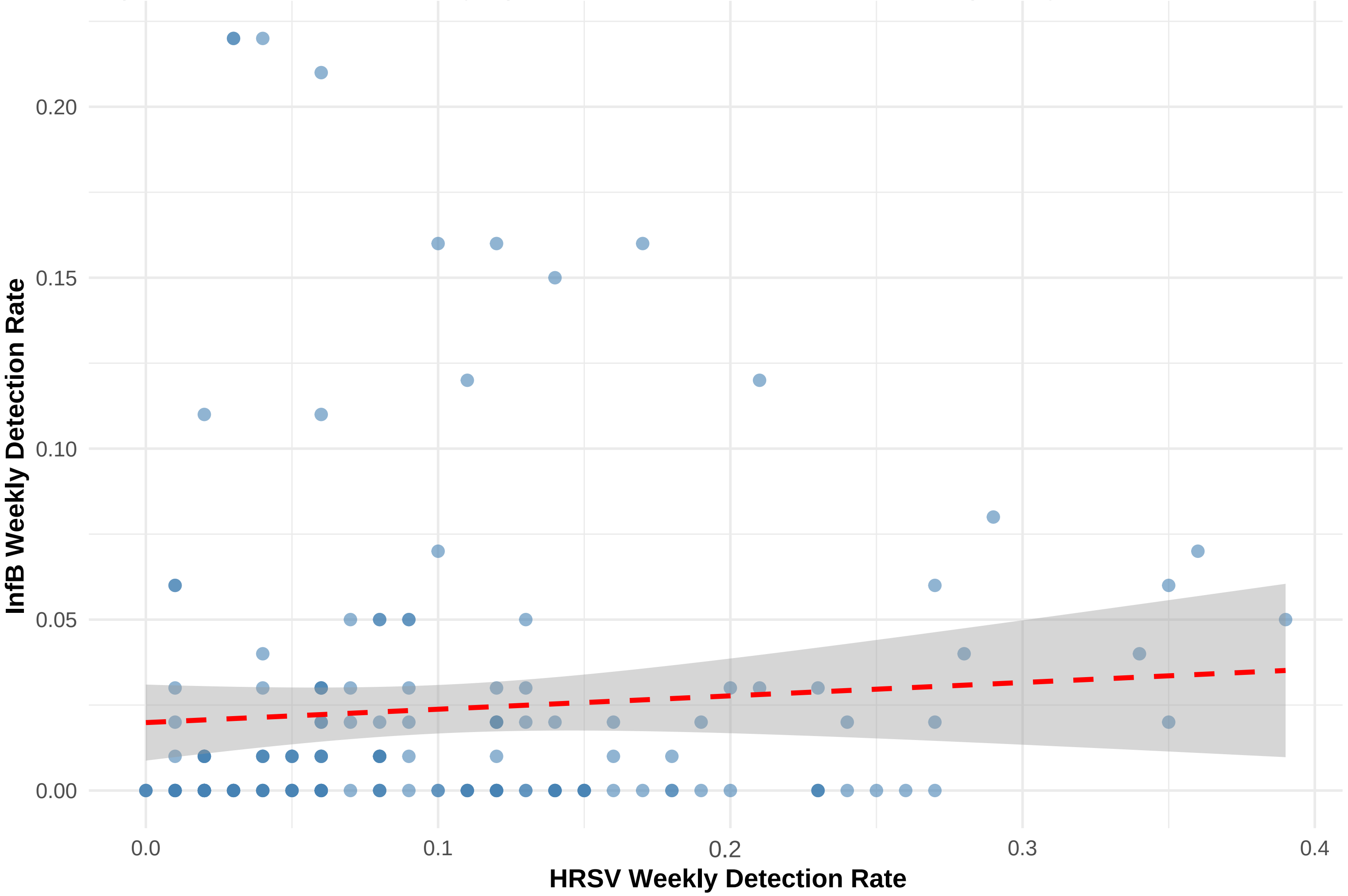

Fig S23 Pathogen Correlation: HRSV vs MP. Scatter plot showing the correlation between the weekly detection rates of HRSV and MP. The Spearman correlation coefficient is -0.18 with a p-value of 0.023, indicating a negative correlation between the two pathogens.

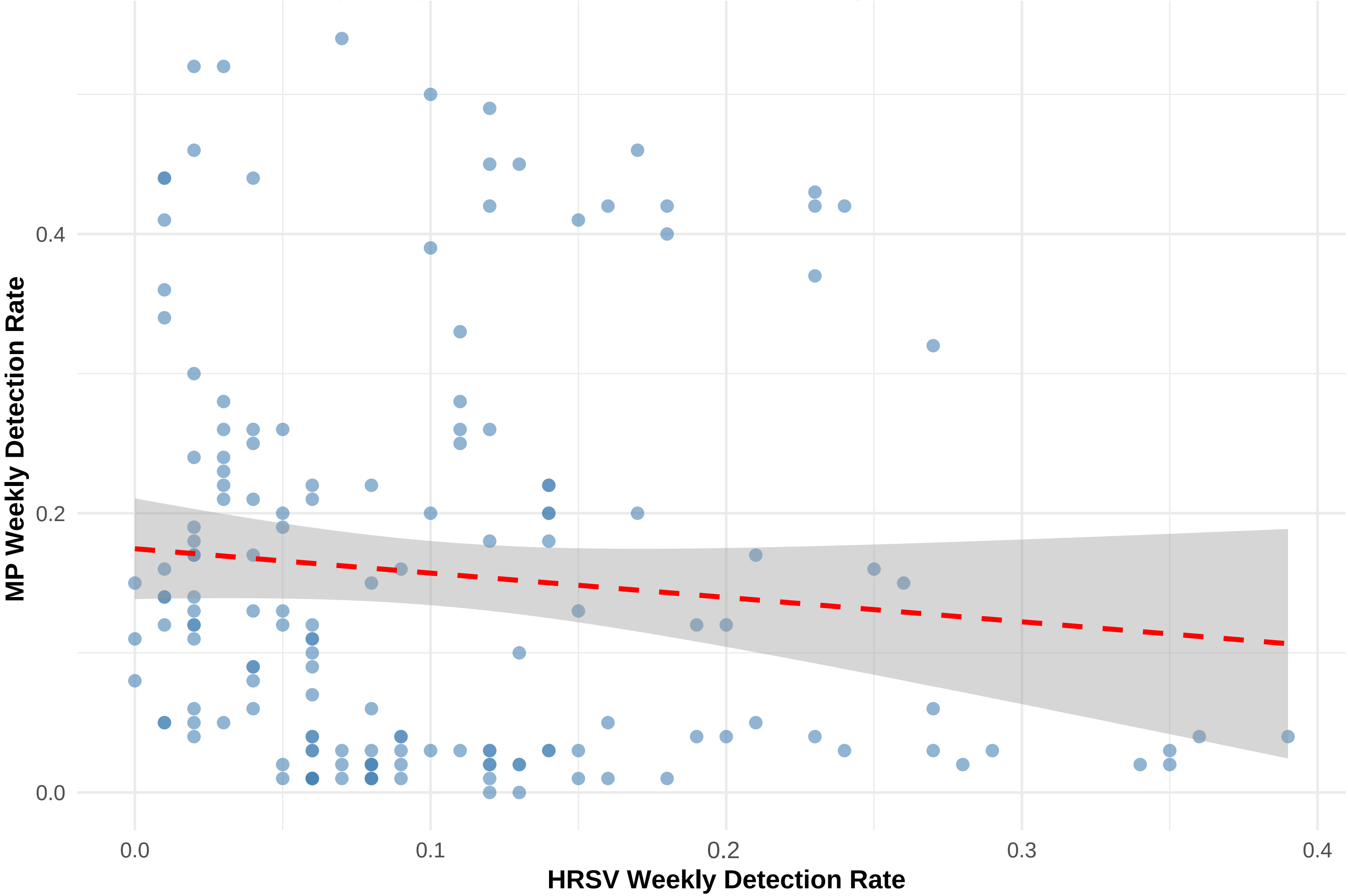

Fig S24 Pathogen Correlation: H1N1 vs H3N2. Scatter plot showing the correlation between the weekly detection rates of H1N1 and H3N2. The Spearman correlation coefficient is 0.334 with a p-value of 1.7e-05, indicating a positive correlation between the two pathogens.

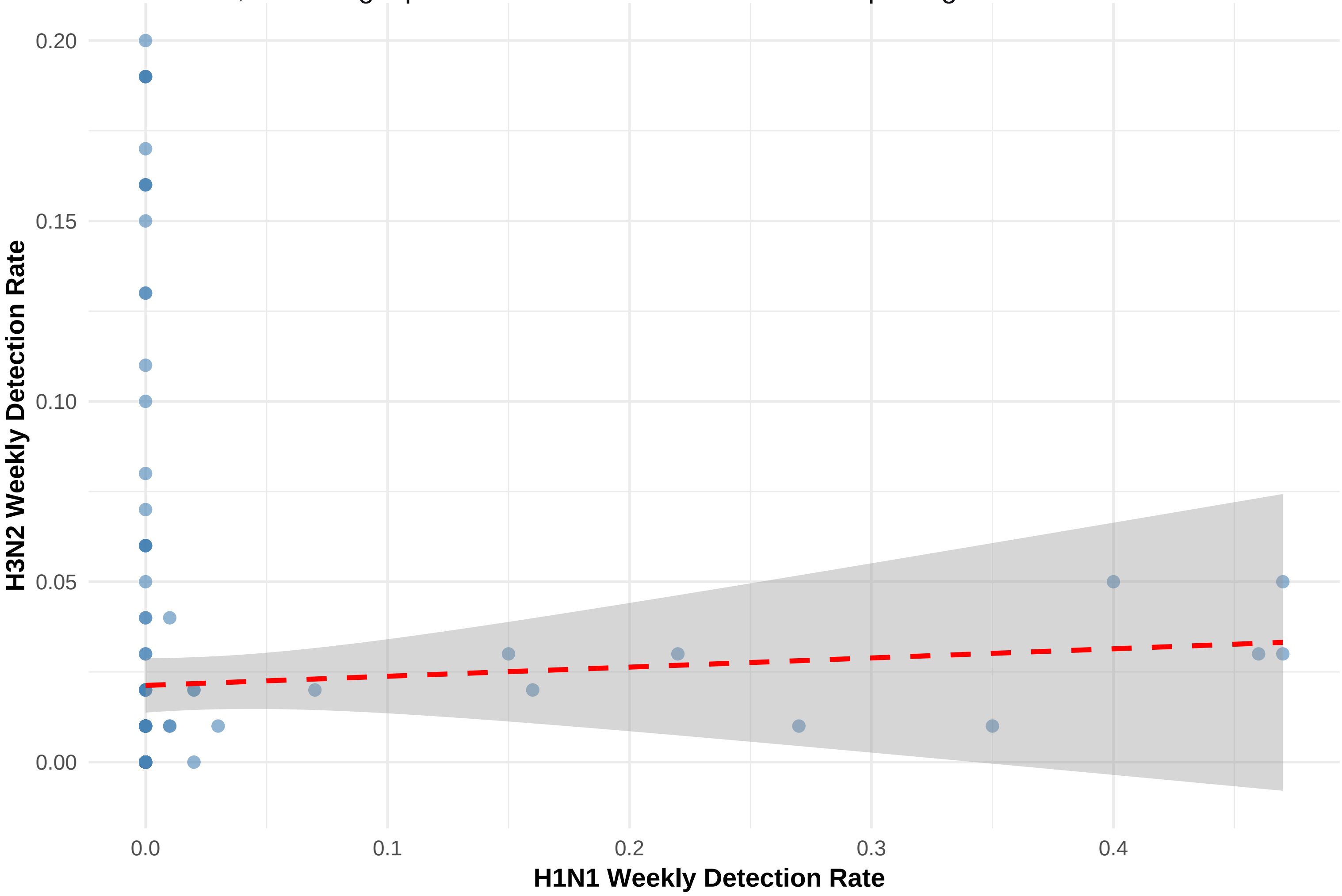

Fig S25 Pathogen Correlation: H1N1 vs InfB. Scatter plot showing the correlation between the weekly detection rates of H1N1 and Influenza B. The Spearman correlation coefficient is -0.22 with a p-value of 0.00526, indicating a negative correlation between the two pathogens.

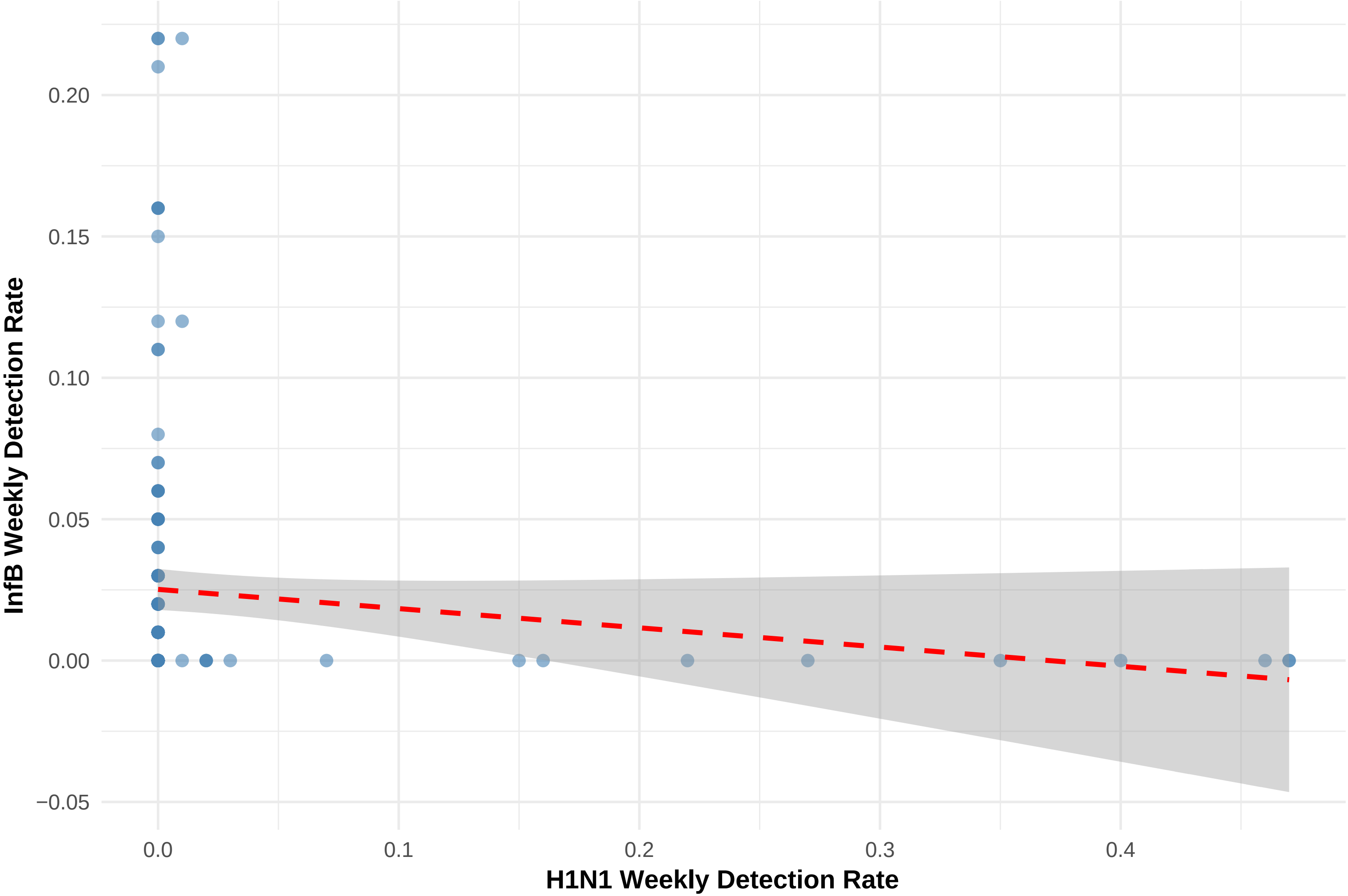

Fig S26 Pathogen Correlation: H3N2 vs HADV. Scatter plot showing the correlation between the weekly detection rates of H3N2 and HADV. The Spearman correlation coefficient is 0.392 with a p-value of 3.11e-07, indicating a positive correlation between the two pathogens.

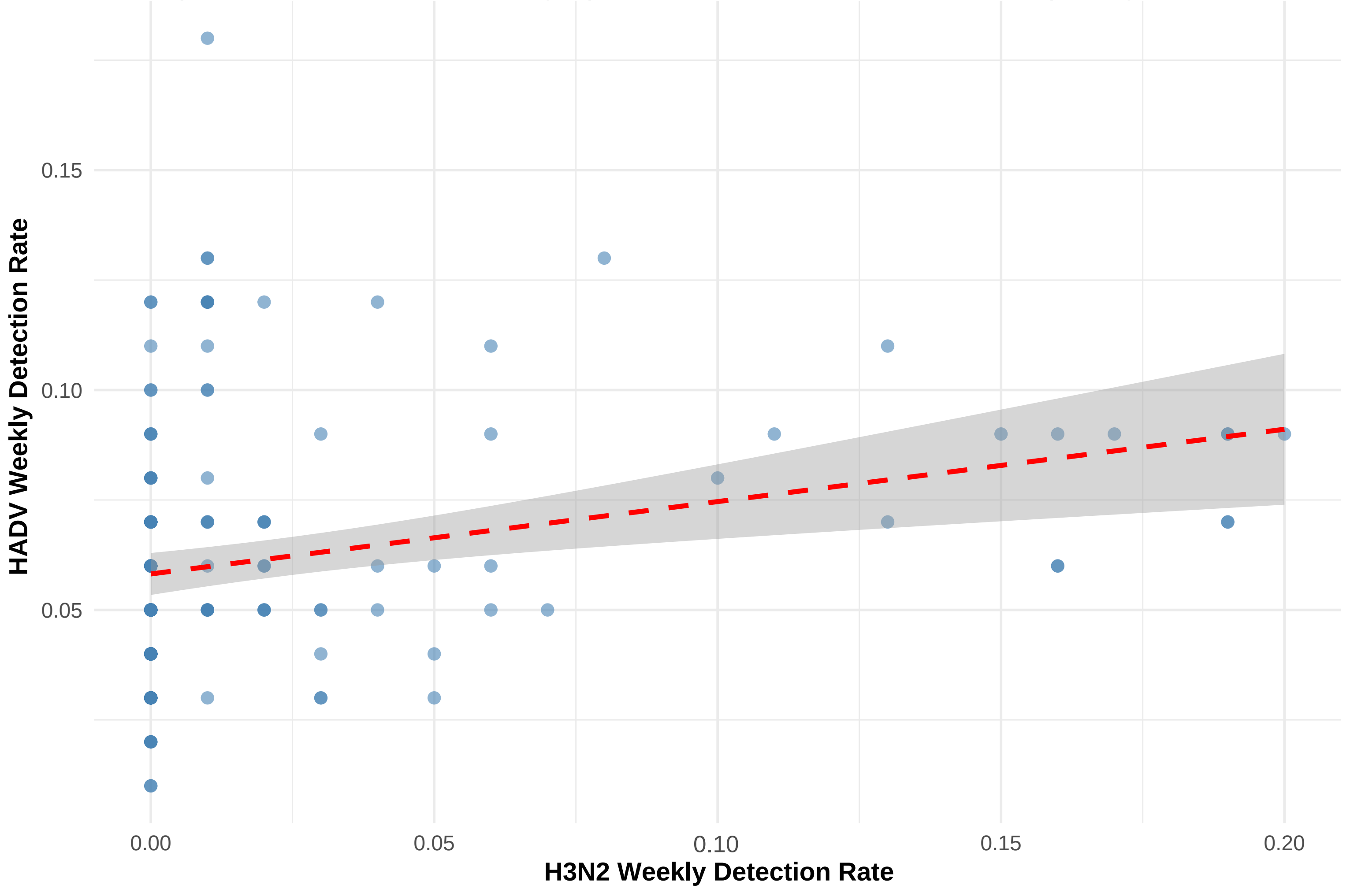

Fig S27 Pathogen Correlation: H3N2 vs MP. Scatter plot showing the correlation between the weekly detection rates of H3N2 and MP. The Spearman correlation coefficient is 0.564 with a p-value of 1.04e-14, indicating a positive correlation between the two pathogens.

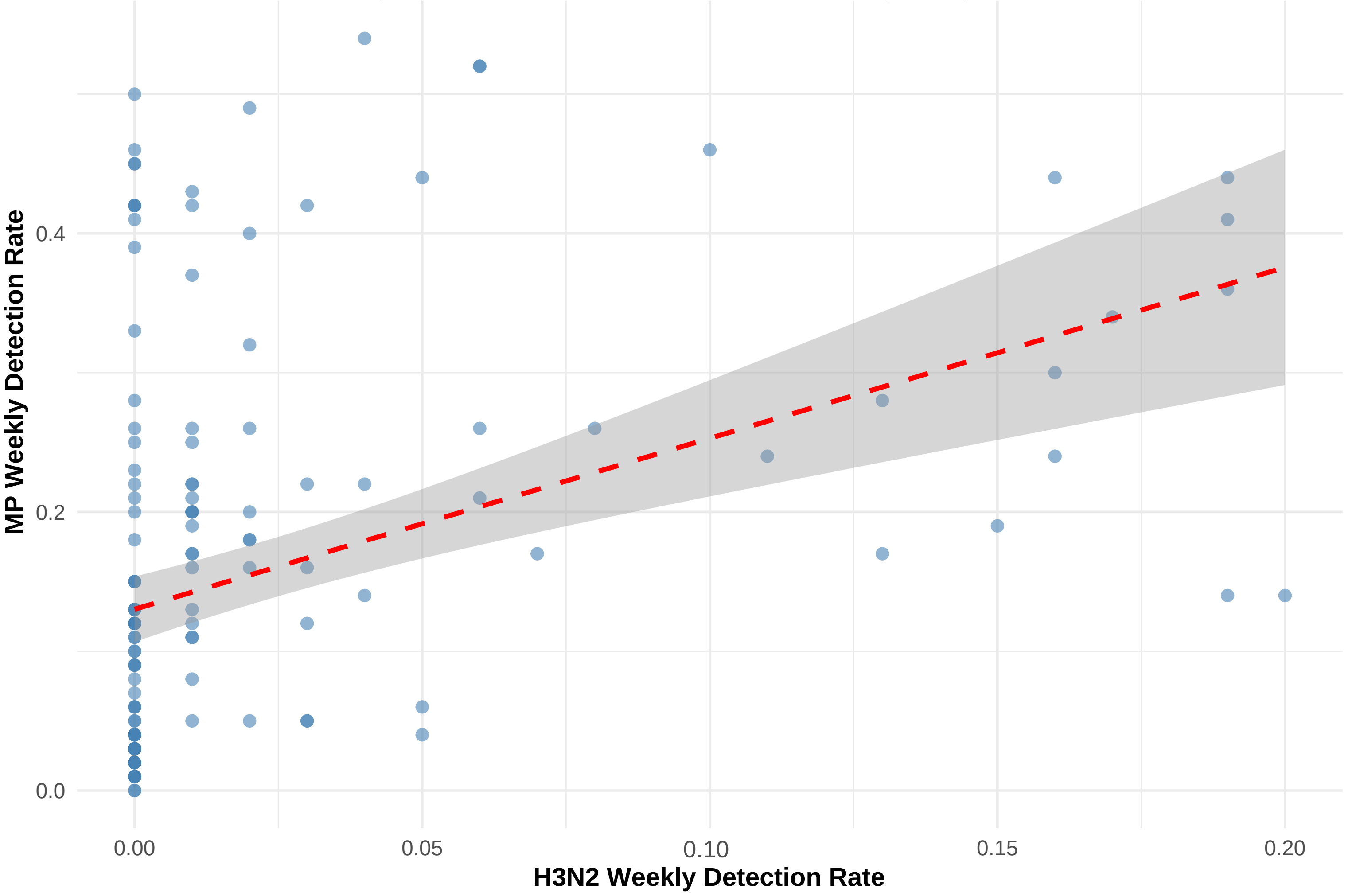

Fig S28 Pathogen Correlation: HMPV vs HADV. Scatter plot showing the correlation between the weekly detection rates of HMPV and HADV. The Spearman correlation coefficient is 0.192 with a p-value of 0.0155, indicating a positive correlation between the two pathogens.

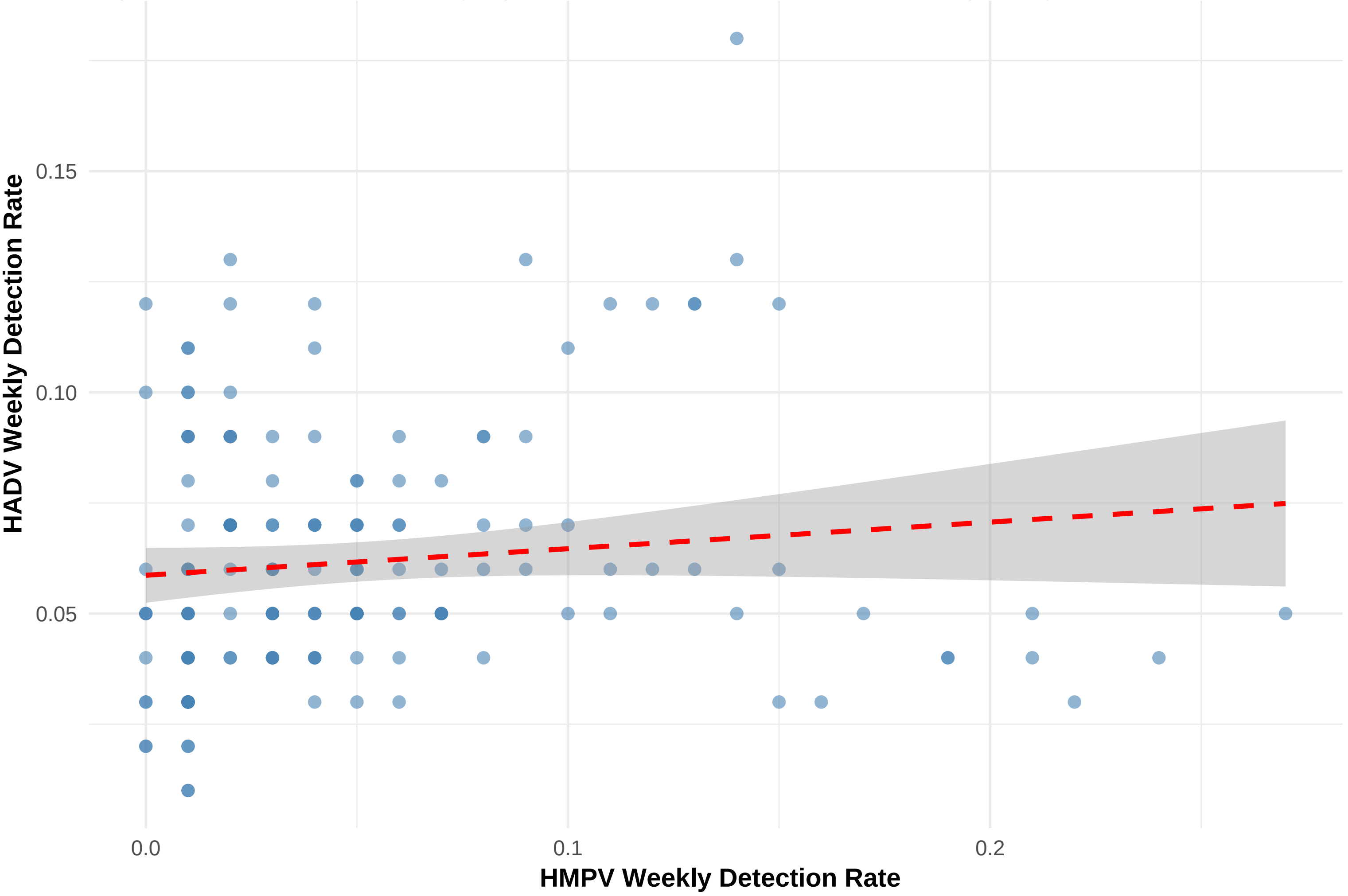

Fig S29 Pathogen Correlation: HMPV vs InfB. Scatter plot showing the correlation between the weekly detection rates of HMPV and Influenza B. The Spearman correlation coefficient is 0.332 with a p-value of 1.93e-05, indicating a positive correlation between the two pathogens.

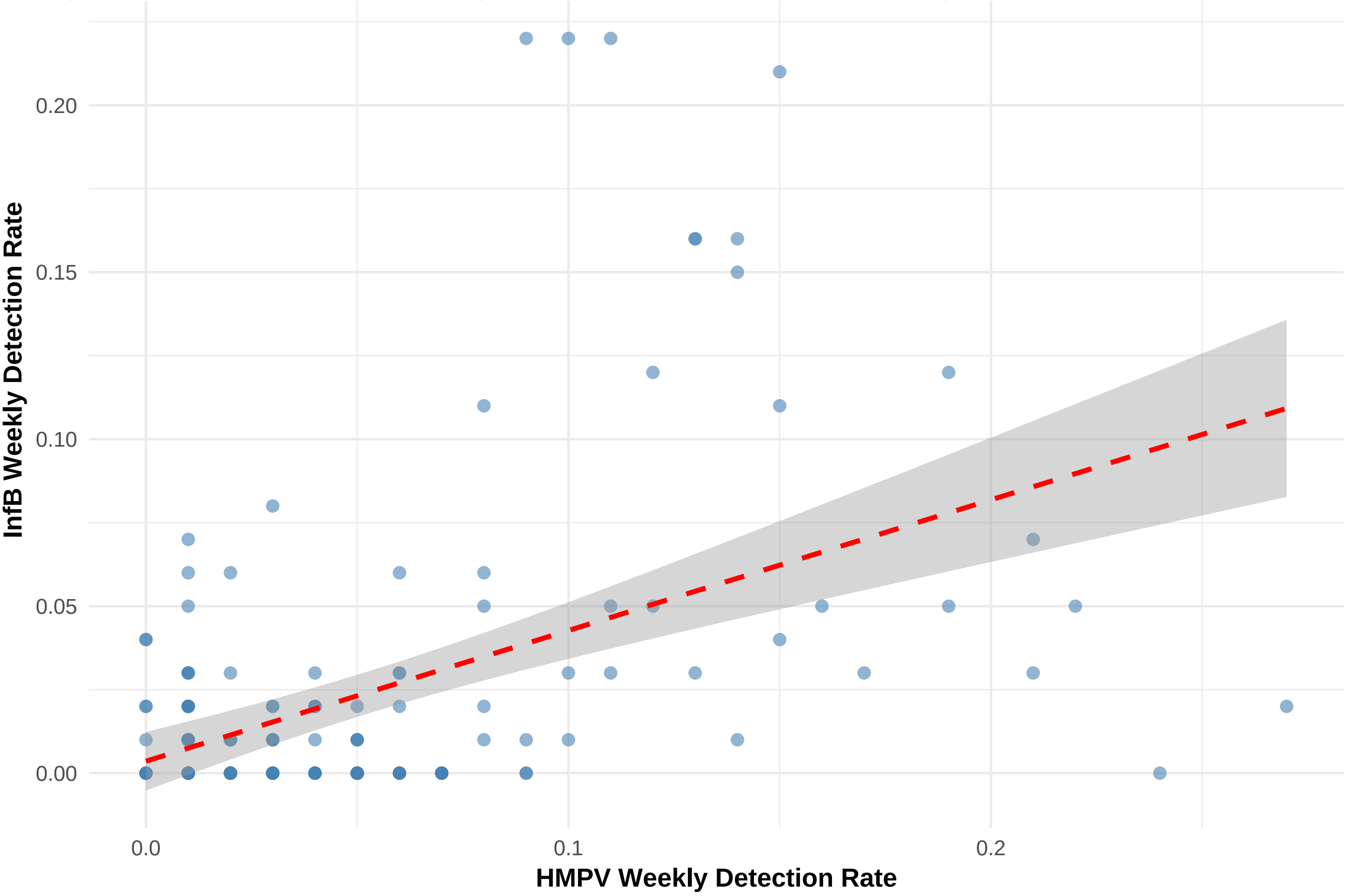

Fig S30 Pathogen Correlation: HADV vs MP. Scatter plot showing the correlation between the weekly detection rates of HADV and MP. The Spearman correlation coefficient is 0.387 with a p-value of 1.96e-05, indicating a positive correlation between the two pathogens.

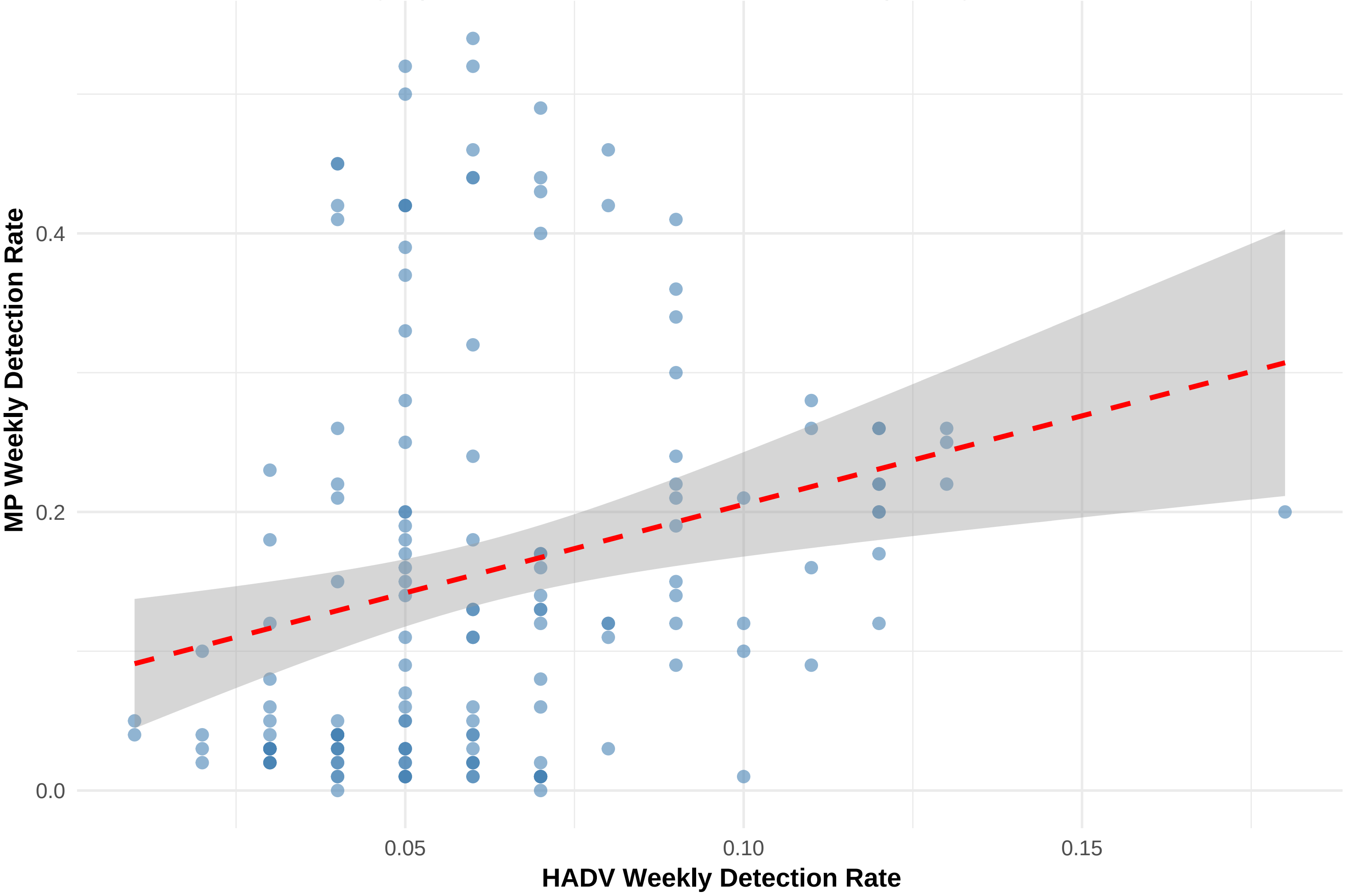

Fig S31 Pathogen Correlation: InfB vs MP. Scatter plot showing the correlation between the weekly detection rates of Influenza B and MP. The Spearman correlation coefficient is -0.262 with a p-value of 1.4e-06, indicating a negative correlation between the two pathogens.

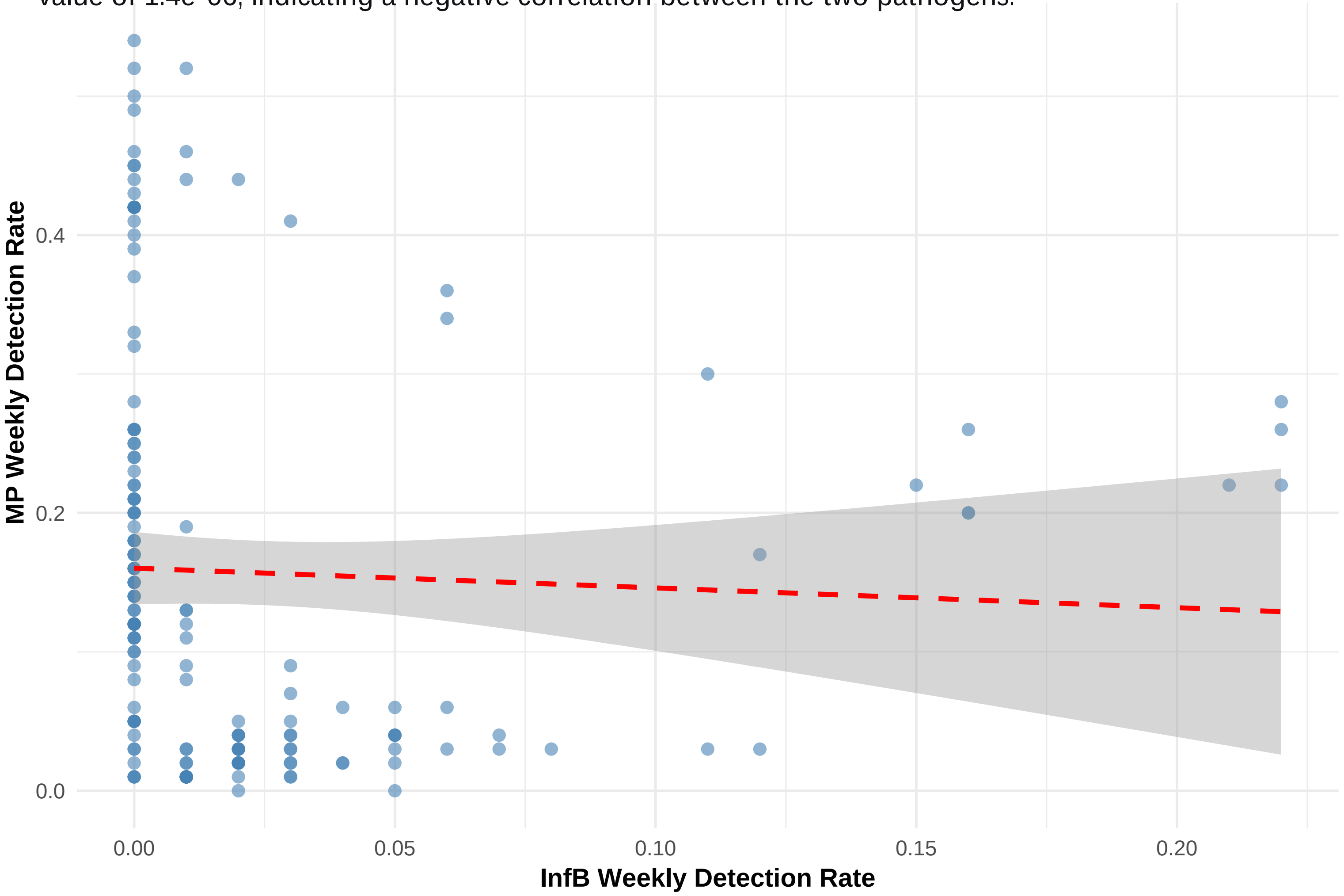

Supplement: Supplementary file 1 — Supporting Information [file PED4-10-47-s001.pdf]
