# Supplementary material for: Interaction analysis of non‐bacterial respiratory pathogens during and after the coronavirus disease 2019 pandemic in two cities along the eastern coast of China
Source: Pediatr Investig. 2026 Jan 19;10(1):47–59. doi: 10.1002/ped4.70034 (PMC12921633; doi:10.1002/ped4.70034)
Supplement: Supplementary file 2 — Supporting Information [file PED4-10-47-s002.pdf]

Supplementary Material for

Interaction analysis of non-bacterial respiratory pathogens during and after the COVID-19 pandemic in two cities along the eastern coast of China

Wanxian Ye, Jishan Zheng, Yungang Yang, Xinyue Song, Xiang Yuan, Lan Yang, Jian Yu, Hailin Zhang, Shunhang Wen

Table S1 Age-group distributions and Chi-square test results for Wenzhou and Ningbo

**Age (years)**	**Wenzhou (*n* = 38 760)**	**Ningbo (*n* = 34 336)**	χ^2	***P*-value**
<1	9202 (23.7)	7607 (22.2)	6.7	0.07
1–3	12 899 (33.2)	12 028 (35.0)		
4–6	8953 (23.1)	7849 (22.8)		
>7	7752 (20.0)	6852 (20.0)		

Table S2 Gender distribution and Chi-square test results for Wenzhou and Ningbo

**Sex**	**Wenzhou (*n* = 38 760)**	**Ningbo (*n* = 34 336)**	χ^2	***P*-value**
Female	18 457 (47.6)	16 587 (48.3)	0.0445	0.8331
Male	20 303 (52.4)	17 749 (51.7)		

Table S3 Confirmed co-infection for each pair of respiratory pathogens across two cities from the 9th week of 2021 to the 9th week of 2024

pathogen1	pathogen2	detection	pathogen1	pathogen2	detection
HRV	MP	1897	HADV	HRSV	129
HADV	MP	428	Boca	HRSV	67
HPIV	MP	307	HMPV	HRSV	65
HRSV	MP	268	HCOV	HRSV	24
H3N2	MP	253	H1N1	HRSV	9
HMPV	MP	180	HADV	HPIV	100
HCOV	MP	174	HMPV	HPIV	72
InfB	MP	126	Boca	HPIV	41
Boca	MP	93	HCOV	HPIV	30
H1N1	MP	27	H3N2	HPIV	15
HRV	InfB	80	H1N1	HPIV	1
HMPV	InfB	54	HADV	HMPV	117
HADV	InfB	51	HCOV	HMPV	30
HRSV	InfB	26	Boca	HMPV	16
HPIV	InfB	19	H1N1	HMPV	5
H3N2	InfB	13	HADV	HCOV	36
Boca	InfB	5	H3N2	HCOV	17
HCOV	InfB	4	Boca	HCOV	9
HRSV	HRV	636	Boca	HADV	67
HADV	HRV	553	H3N2	HADV	56
HPIV	HRV	527	H1N1	HADV	19
HMPV	HRV	333	Boca	H3N2	8
Boca	HRV	269	H1N1	H3N2	2
H3N2	HRV	138	HADV	H1N1	19
HCOV	HRV	106	Boca	H1N1	7
H1N1	HRV	59	HPIV	H1N1	1
HPIV	HRSV	154	H3N2	Boca	8

Table S4 Estimation results for equation HRSV

**Variable**	**Estimate**	**Std. Error**	***t***	**Pr (>|t|)**
HRV	−0.031	0.059	-0.523	0.602
Boca	0.084	0.195	0.429	0.668
HPIV	0.377	0.143	2.631	0.009
HCOV	0.014	0.276	0.052	0.959
HRSV	0.014	0.082	0.166	0.868
H1N1	−0.118	0.107	−1.105	0.271
H3N2	−0.107	0.181	−0.590	0.556
HMPV	0.189	0.105	1.807	0.073
HADV	0.124	0.122	1.015	0.312
InfB	−0.196	0.141	−1.386	0.168
MP	−0.102	0.082	−1.234	0.219
const	0.001	0.002	0.367	0.714
